# Inverse design of all-dielectric metasurfaces with accidental bound states in the continuum

**DOI:** 10.1515/nanoph-2023-0373

**Published:** 2023-09-22

**Authors:** Sergei Gladyshev, Theodosios D. Karamanos, Lina Kuhn, Dominik Beutel, Thomas Weiss, Carsten Rockstuhl, Andrey Bogdanov

**Affiliations:** Institute of Physics, University of Graz, Universitätsplatz 5, 8010 Graz, Austria; Institut Langevin, ESPCI Paris, Université PSL, CNRS, 75005 Paris, France; Institute of Theoretical Solid State Physics, Karlsruhe Institute of Technology, 76131 Karlsruhe, Germany; Steinbuch Centre for Computing – Scientific Computing & Mathematics, Karlsruhe Institute of Technology, Karlsruhe, Germany; Institute of Nanotechnology, Karlsruhe Institute of Technology, 76344 Eggenstein-Leopoldshafen, Germany; Qingdao Innovation and Development Center of Harbin Engineering University, Qingdao 266000, Shandong, China; 4th Physics Institute and SCoPE, University of Stuttgart, Pfaffenwaldring 57, D-70569 Stuttgart, Germany; Harbin Engineering University, Harbin, 150001, China

**Keywords:** inverse design, bound states in the continuum, metasurfaces, T matrix, machine learning, multipole approximation

## Abstract

Metasurfaces with bound states in the continuum (BICs) have proven to be a powerful platform for drastically enhancing light–matter interactions, improving biosensing, and precisely manipulating near- and far-fields. However, engineering metasurfaces to provide an on-demand spectral and angular position for a BIC remains a prime challenge. A conventional solution involves a fine adjustment of geometrical parameters, requiring multiple time-consuming calculations. In this work, to circumvent such tedious processes, we develop a physics-inspired, inverse design method on all-dielectric metasurfaces for an on-demand spectral and angular position of a BIC. Our suggested method predicts the core–shell particles that constitute the unit cell of the metasurface, while considering practical limitations on geometry and available materials. Our method is based on a smart combination of a semi-analytical solution, for predicting the required dipolar Mie coefficients of the meta-atom, and a machine learning algorithm, for finding a practical design of the meta-atom that provides these Mie coefficients. Although our approach is exemplified in designing a metasurface sustaining a BIC, it can, also, be applied to many more objective functions. With that, we pave the way toward a general framework for the inverse design of metasurfaces in specific and nanophotonic structures in general.

## 1 Introduction

Bound states in the continuum (BICs) are non-radiating solutions to the wave equation with a spectrum embedded in the continuum of the propagating modes in the surrounding space. BICs are a general wave phenomenon that can exist in a variety of acoustic, hydrodynamic, quantum mechanical, and electromagnetic systems [1–3]. Because of their infinite radiative lifetimes, BICs are actively studied in optics and photonics, opening up enormous opportunities to realize compact planar high-Q metastructures necessary for biosensing, integrated nonlinear nanophotonics, and an enhancement of light–matter interactions [4]. In photonics, the most promising platform supporting BICs are *metasurfaces* [5]. Metasurfaces offer a vast number of designs with materials compatible with BIC. In particular, metasurfaces with BICs demonstrated their efficiency for lasing [6–10], biosensing [11–14], enhancing nonlinear optical effects [15–18] and polaritons [19–22], leading, recently, to the first experimental demonstration of room-temperature exciton–polariton condensation from a BIC [23].

Optical resonances in periodic metasurfaces radiate only into the open diffraction channels, while BICs remain non-radiating due to the vanishing coupling to all open diffraction channels. For subwavelength metasurfaces, there is only one open diffraction channel and, thus, one coupling coefficient. It can vanish due to symmetry reasons or due to a fine-tuning of the system’s geometrical or material parameters [24]. In the first case, the BICs are called *symmetry-protected* and usually exist in high symmetry points of the *k*-space. In the second case, BICs are called *accidental* or *parametric*, and they can exist at an arbitrary point in the *k*-space along the high-symmetry directions [25]. This explains the term “accidental”. The accidental BICs are extremely sensitive to changes in the geometry of the unit cell or the material parameters. Such a high sensitivity makes it challenging and time-consuming to design metasurfaces with a pre-required or fixed spectral and angular position of BIC.

As an expansion to the time-consuming and more classic optimization approach using analytical formulation tools [26, 27], an *inverse design* approach can be used [28–33]. The term “inverse design”, herein, refers to the process of designing a metasurface with optical properties by initially specifying the desired response rather than the structure of the metasurface itself. Such an inverse design involves dedicated algorithms, e.g., from Bayesian inference, topology optimization, or artificial neural networks. The purpose is always to find the optimal metasurface that provides a predefined optical response [34–37]. Inverse design can offer metasurfaces with a wide range of optical properties, including phase shifts, polarization conversions, and beam steering. It is a powerful tool for designing advanced optical devices and has applications in fields such as imaging, sensing, and telecommunications [38–41]. Despite the power of inverse design methods based on artificial neural networks, the underlying physics of the found optimum often remains vague.

**Figure 1:**
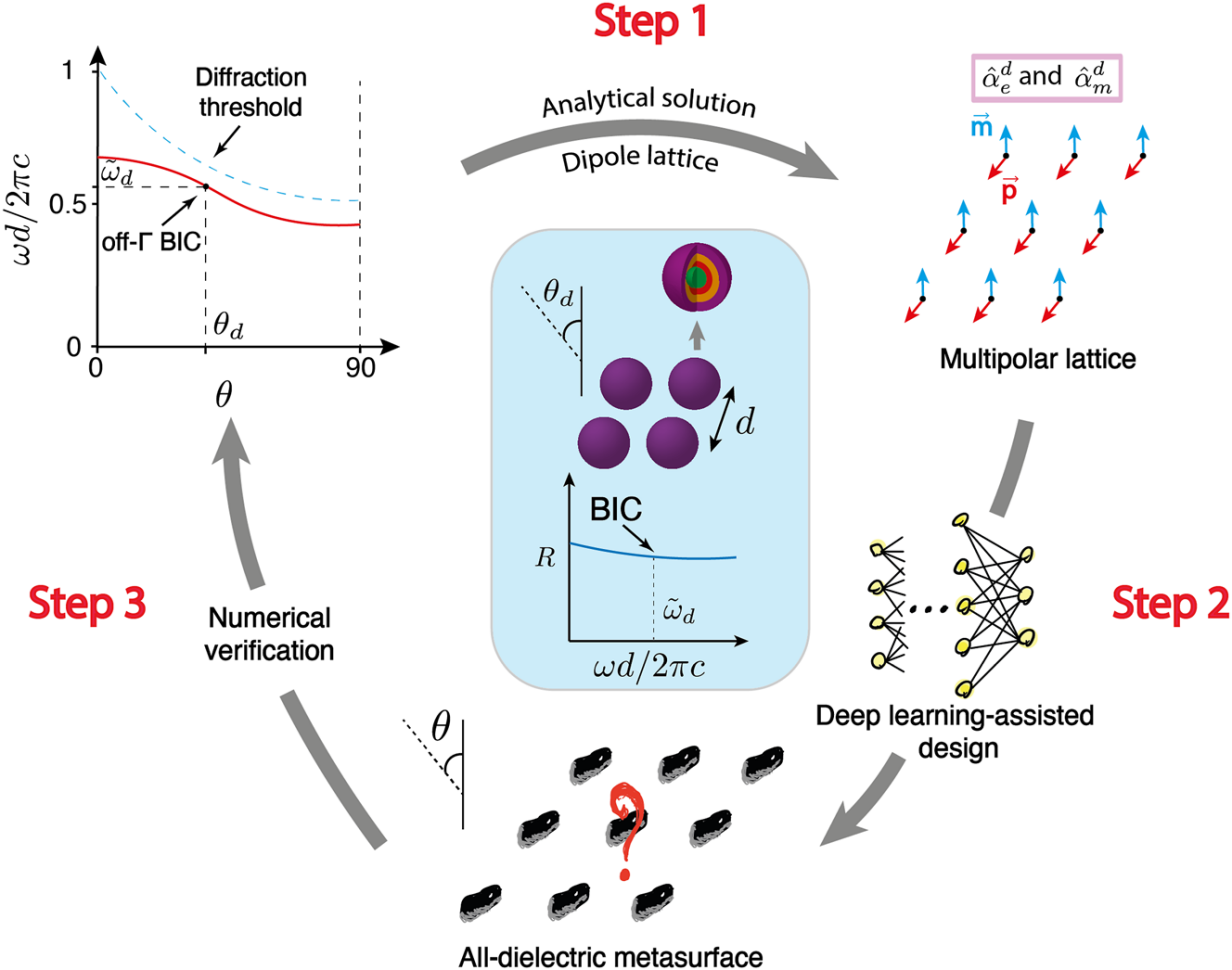
The concept of our proposed framework. Beginning from the top left, an accidental BIC sustained by a metasurface is desired at a specific normalized frequency and incidence angle given by $\left({\tilde{\omega }}_{d},\theta_{d}\right)$ . Then, the metasurface is analytically modeled via the multipolar expansion up to dipolar order. The predefined BIC is supported when the isotropic meta-atom decorating the unit cell of the metasurface has a specific electric and magnetic dipolar polarizability. Following that, a realistic core–shell particle that provides the desired polarizabilities is designed via a machine-learning algorithm in the second step. Finally, the proposed, realistic metasurface is numerically validated concerning its performance and robustness in the third step. By the latest at that stage, a realistic metasurface is described in all its details.

In this work, we develop a physics-inspired inverse design procedure for all-dielectric metasurfaces sustaining an accidental BIC at a predefined frequency and incidence angle. Our framework is based on a smart combination of a semi-analytical approach and a dedicated machine-learning algorithm. Our procedure includes three steps schematically shown in Figure 1. In the first step, the problem is solved within a “toy” model. Here, we consider all-dielectric meta-atoms in dipole approximation and capture their response using a T-matrix. After representing the meta-atoms utilizing the electric/magnetic (dipole) polarizabilities, or, equivalently, the scattering (dipole) Mie coefficients, the metasurface model is set up. The existence condition for an accidental BIC at a predefined spectral position and angle of incidence is derived analytically as a function of the Mie coefficients of the constituting particle through the identification of the system’s eigenmodes. At the end of that first step, we know the polarizabilities of the particle such that the metasurface offers the predefined BIC. In the second step, using a dedicated artificial neural network, we identify the geometrical and material parameters of a core–shell, spherical particle that offers the desired and previously identified polarizabilities so that the metasurface sustains the predefined BICs. The neural network accounts for the practical limitations on the sizes of the spherical particle shells and refractive indices of the materials. In this way, a physically feasible meta-atom is found that provides the desired BIC when placed on an infinite 2D array. In the last step, we numerically verify the design. We also test the robustness of the designed BIC position in *k*-space if higher-order multipoles contribute besides the dipolar one, namely quadrupoles and octupoles. This entire process is described in the following.

## 2 Results

### 2.1 Step 1: BIC in dipolar metasurface

**Figure 2:**
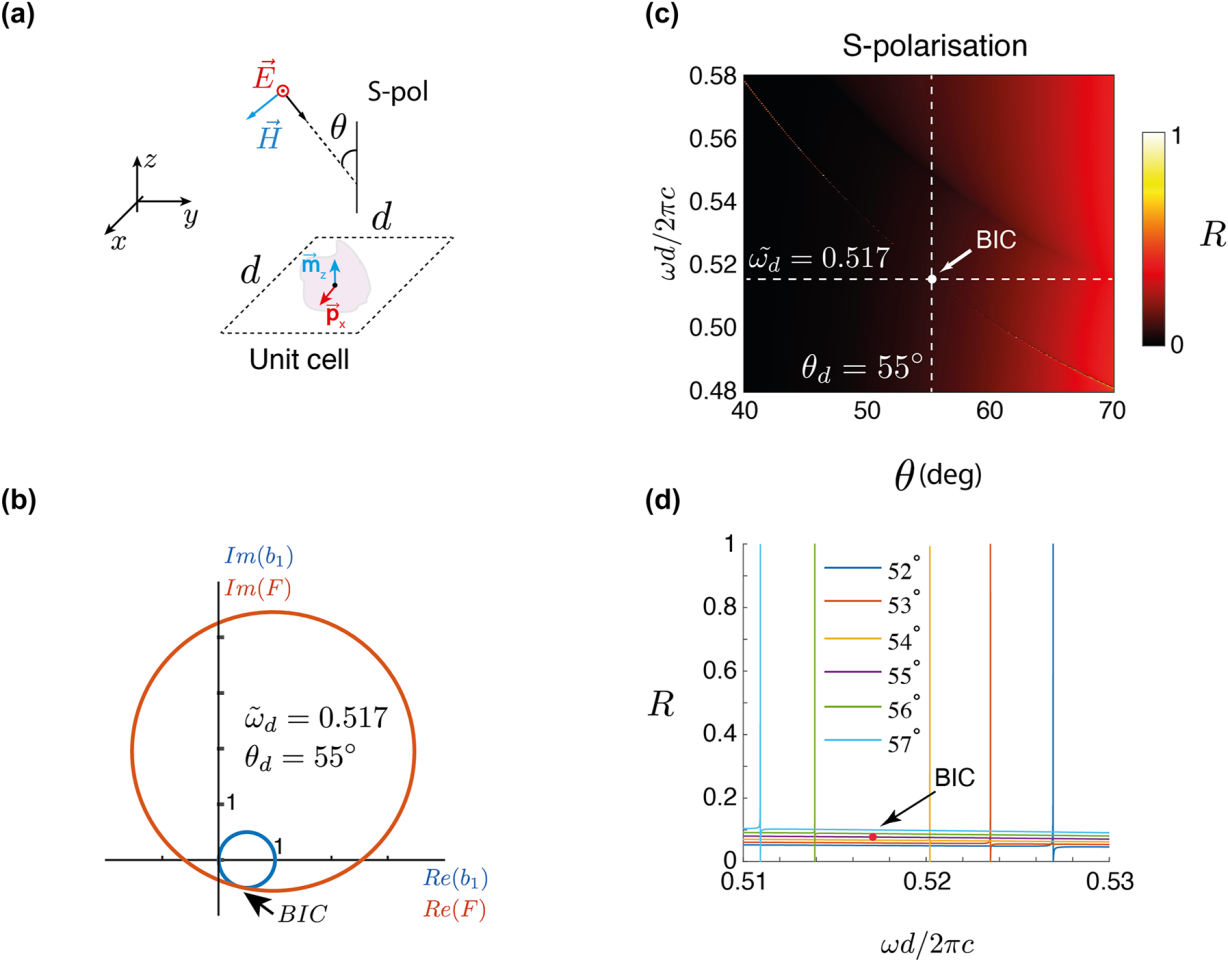
The toy model: (a) a unit cell of a metasurface made from a periodic arrangement of scatterers with electric and magnetic dipole polarizabilities in a square lattice. (b) A graphical representation of how the Mie coefficients are identified that leads to the desired response. The analytical expression for the Mie coefficient that needs to be satisfied to sustain a BIC at a predefined normalized frequency and incidence angle for a given period is plotted separately concerning its left- and right-handed sides. Each side depends only on one Mie coefficient. By parametrizing the Mie coefficients with the Mie angles, both sides of the expression in the complex plane have been plotted. From the point of crossing, the Mie coefficients that provide the desired BIC are identified. (c) The reflection *R* as a function of dimensionless frequency *ωd*/2π*c* and angle of incidence *θ* for a square lattice with period *d* = 450 nm. The lattice is decorated with particles that offer the previously identified Mie coefficients. The appearance of the BIC at the predefined frequency and incidence angle can be seen. (d) The reflection *R* as a function of dimensionless frequency *ωd*/2π*c* in close proximity to the off-Γ BIC.

Let us consider in our “toy” model a metasurface consisting of isotropic and non-absorbing particles described in dipole approximation. The particles are arranged on a square lattice with a subwavelength period, i.e. for any incidence angle, there is only a zeroth diffraction order. The considered unit cell is shown in Figure 2(a). The surrounding medium is a vacuum. The metasurface is illuminated by a time-harmonic linearly polarized plane wave in either TE (or *s*) or TM (or *p*) polarization.

Considering the renormalization of the particle’s polarizability due to the lattice interaction and imposing a condition that expresses the existence of a resonance, i.e., a denominator in the renormalized polarizability has to be zero, allows for the analytical identification of an equation that must be satisfied for the existence of a BIC. For a given lattice, frequency, polarization, and incidence angle, this equation expresses the necessary relation between the dipolar magnetic *b*_1_ and electric *a*_1_ Mie coefficients to encounter a BIC. A derivation is given in the METHODS section, but the final expressions read

(1a) $$b_{1}=-\frac{1+a_{1}\left(C_{1}+C_{3}\right)}{C_{2}+a_{1}\left(C_{1}C_{2}+C_{2}C_{3}-2\;C_{5}^{\;2}\right)}=F\left(a_{1}\right),$$

(1b) $$a_{1}=-\frac{1+b_{1}\left(C_{1}+C_{3}\right)}{C_{2}+b_{1}\left(C_{1}C_{2}+C_{2}C_{3}-2\;C_{5}^{\;2}\right)},$$

where (1a) and (1b) refer to a TE/*s*-polarized or a TM/*p*-polarized incidence, respectively. The equations above are analogous to the equations found in [26, 27], but, now, we utilize the versatile Mie coefficients of the particles under study. The *C*_*i*_ (*i* = 1, …, 5) are the elements of the lattice interaction coefficients matrix ${\bar{\bar{C}}}_{s}$ (see Supplementary Material, Section S5). These interaction coefficients are calculated via an Ewald summation methods [42–44].

The elements of ${\bar{\bar{C}}}_{s}$ depend on the lattice constant *d*, frequency *ω*, and Bloch wavevector. Therefore, they carry the information about the desired point of operation. Once they are fixed, the Mie coefficients can be identified such that a BIC is supported. Note that the equations for *s*- and *p*-polarization are similar. Only the coefficients *a*_1_ and *b*_1_ are swapped, as anticipated, due to symmetry. One should notice from (1) that the electric-magnetic lattice coupling coefficient is crucial for the existence of a simultaneous solution for (*a*_1_, *b*_1_), indicating the importance of multipolar, electromagnetic coupling to realize BICs. The analytical derivation of Eq. (1) is further elaborated in the Supplementary Material.

Hence, for the specific scenario considered, Eq. (1) provides the exact condition to encounter a BIC for *s*- or *p*-polarized incidences, respectively. To further simplify the design, the Mie coefficients are parametrized using what is called the Mie angles [45]. Note that we consider a system that possesses a time-reversal symmetry $\varepsilon \left(\vec{r}\right)=\varepsilon^{*}\left(\vec{r}\right)$ , since one of the most important conditions for obtaining a perfectly confined state, which also leads to a reduction of degrees of freedom [25]. For a lossless particle, a single angle bound between −π/2 and π/2 is sufficient to express any possible value a Mie coefficient might attain. A representation of the Mie coefficients in terms of these Mie angles is highly beneficial for the further design. For example, for the s-polarized incidence case, by substituting dipole electric and magnetic Mie angles, *θ*_E1_ and *θ*_M1_ (see METHODS) into Eq. (1a), the exact solution can be easily obtained via a non-linear equation solver for a given wavelength, incidence angle, and lattice dimension.

The process of identifying the BIC graphically can be demonstrated by assuming an *s*-polarized incidence, as depicted in Figure 2(a). After expressing the right hand side of Eq. (1a) as *F*(*a*_1_). All possible Mie angles in the range of [−π/2, π/2], which parametrize the magnetic and electric dipolar coefficients, are swept through. The left-hand side (only the *b*_1_ coefficient) and the right-hand side of Eq. (1a) are shown in the complex plane in Figure 2(b). The left-hand side, i.e. only the *b*_1_ coefficient, and the right-hand side of (1a) are shown in the complex plane in Figure 2(b). As required by lossless scatterers, all possible Mie coefficients, including the ones of *b*_1_, lie on a circle in the complex plane with a center at the (0.5, 0) point and a radius of 1. It can be identified that the BIC is located where Re{*b*_1_} = Re{*F*(*a*_1_)} and Im{*b*_1_} = Im{*F*(*a*_1_)} for a specific wavelength, incidence angle, and lattice constant. Once the *b*_1_ coefficient is known, the *a*_1_ value can be explicitly calculated. If there were losses in the system, then in the expressions *a*_1_ and *b*_1_ (see METHODS) additional terms $\tan\theta_{\text{E}1,M1}^{\prime }$ appeared in the denominator (see Supplementary Material), then there would be no intersection points of the circles *b*_1_ and *F*(*a*_1_) in the complex plane in Figure 2(b), indicating the nonexistence of BIC in such a system.

As an example, we find a combination of Mie angles (*θ*_*E*1_, *θ*_*M*1_), or, in other words, ${\bar{\bar{T}}}_{0}$ matrix, for which a BIC exists for the desired normalized frequency ${\tilde{\omega }}_{d}=\omega \;d/2\pi c=0.517$ and incident angle *θ*_d_ = 55° in TE-/*s*-polarization. The reflection from a metasurface formed by the 2D array of dipolar particles, corresponding to the calculated (*a*_1_, *b*_1_) values, is shown in Figure 2(c). The reflection is shown as a function of the normalized frequency, *ω d*/2π*c*, and the incidence angle, *θ*. Additionally, we have been assuming here that the T-matrix is non-dispersive.

For the selected *a*_1_ and *b*_1_ dipole moments, a narrow resonant band is formed. This resonant band disappears precisely at the target parameters $\left({\tilde{\omega }}_{d},\theta_{d}\right)$ , thus, hailing a BIC. To make the success of the proposed methodology more explicit, in Figure 2(d), we illustrate the change in resonance shape for the reflection coefficient, *R*, as a function of *ωd*/2π*c*, for different incident angles *θ* from 52° to 57°. The resonance becomes infinitely narrow at an angle of *θ*_d_ at ${\tilde{\omega }}_{d}$ , or the *Q*-factor becomes infinite. It implies that the incident wave does not interact with the metasurface that gets fully transparent. Therefore, the presented methodology succeeds in providing the Mie coefficients for a spherical particle to observe a BIC at a specific frequency and an angle of incidence of a plane wave upon a periodic arrangement of the particle.

### 2.2 Step 2: Deep learning-assisted engineering of spherical particles for BIC realization

In the previous subsection, a method was presented that provides the T-matrix for an isotropic particle to achieve an accidental BIC for a specific normalized frequency and incidence angle. Although this approach was successful, if one wants to provide a practical design of a metasurface that exhibits BICs, the calculated (*a*_1_, *b*_1_) values must be linked to a realistic scatterer. In this work, we employ a deep-learning scheme to assign the calculated Mie coefficients to a spherical core–shell nano-particle of realistic dimensions and made from existing materials for an operation at optical wavelengths.

**Figure 3:**
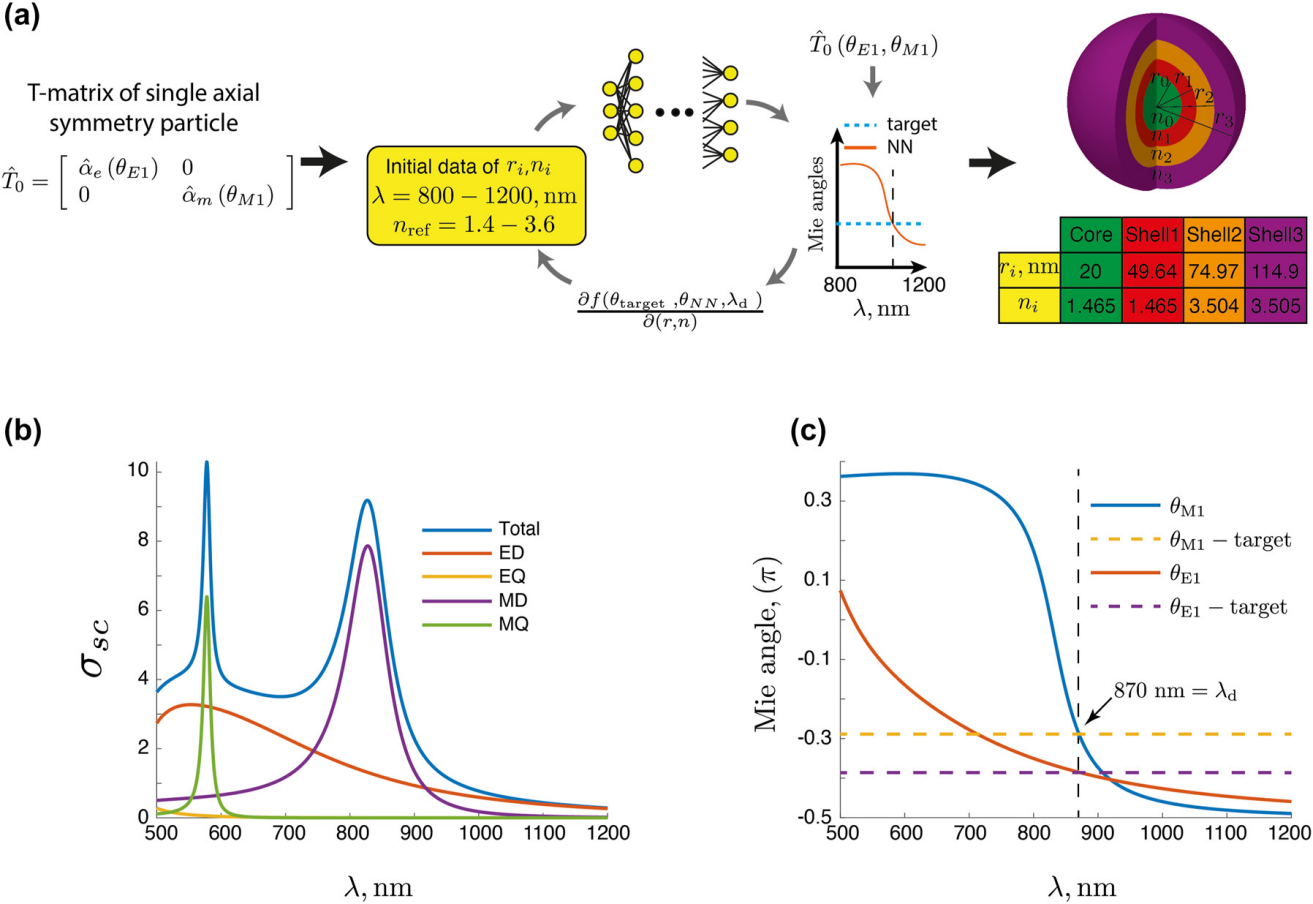
Inverse design: (a) scheme to find the geometrical and material parameters of a meta-atom (core–shell particle) with a target optical response. In the central part, there is a fully differentiable artificial neural network that can predict the Mie angles for a given core–shell particle. The network was trained within a given spectral region using discrete material classes and constrained geometrical dimensions for the core and the shell that make the design feasible for realization. A gradient descent is then used to identify the parameters characterizing the core–shell particle such that predefined dipolar Mie angles are provided at a design wavelength. At the very end, a second optimization is performed where the refractive indices of the considered materials are fine-tuned to reach an absolute precision. The table shows the design parameters for the example considered in the text. (b) Contribution of each multipole moment (up to quadrupolar order) to the scattering cross-section of the core–shell particle *σ*_*sc*_ as a function of the wavelength *λ* for the final design. (c) Mie angles as a function of wavelength: the dotted line refers to the Mie angles of the optimized toy model, and the solid line refers to the Mie angles of the final design. The intersection of the dotted and solid lines in the *λ*_d_ = 870 nm.

To find a suitable scatterer that provides desired Mie angles, a gradient-based deep learning-assisted approach is performed, schematically explained in Figure 3(a). More details on the Artificial Neural Networks (ANNs) can be found in the article [46]. Initially, we train a set of ANNs to predict the Mie angles of coated dielectric spheres made from one to five shells and at 200 distinct wavelengths, between 800 nm and 1200 nm. A part of the ANNs is also a classifier that predicts the most probable number of shells to provide the requested Mie angles. Actually, the exemplarily shown core–shell particle in Figure 3(a) has only three shells. Our trained ANN simply predicted at the end a core–shell particle with three shells only as the most appropriate, which is illustrated here. The materials of the layers are restricted to discrete refractive indices classes ranging from 1.4–3.5, values that are realistic at optical wavelengths for specific materials (see Table 1). Note, that our goal, here, is to design a metasurface exhibiting a BIC at a specific frequency without examining what happens at neighboring frequency regions. Therefore, there is no need to include material dispersion and we utilize fixed real values of refractive indexes. Nevertheless, these values used in this work correspond to possible values of typical materials used in photonics, like GaAs, SiO_2_ etc. The radius of the core is limited to 20 nm–50 nm. The dimension of each shell is restricted to 20 nm–40 nm. The ANNs are used as a fully differentiable surrogate model in a gradient-based optimization algorithm, namely the limited-memory Broyden–Fletcher–Goldfarb–Shanno algorithm including boundary constraints, or L-BFGS-B [47].

**Table 1:** Diferent materials, associated classes and refractive indices.

Class	1	2	3	4	5	6	7
Refractive index	1.4649	1.7196	1.9447	2.0745	2.4317	3.0	3.5
Material	SiO_2_	MgO	ZnO	ZrO_2_	TiO_2_	AlAs	GaAs

**Table 2:** The classification of modes by irreducible representations and the multipole composition of the eigenmode for *C*_2*v*_ symmetry group in direction Γ*X* in the dipole approximation for different bases.

**Irreducible representation**	***A*_1_**	***A*_2_**	***B*_1_**	***B*_2_**
Cartesian basis	*p*_*y*_	*m*_*y*_	*p*_*z*_, *m*_*x*_	*m*_*z*_, *p*_*x*_
Spherical basis	$a_{11}^{\text{e}}$ , $a_{1-1}^{\text{e}}$	$a_{11}^{\text{m}}$ , $a_{1-1}^{\text{m}}$	$a_{10}^{\text{e}}$ , $a_{11}^{\text{m}}$ , $a_{1-1}^{\text{m}}$	$a_{10}^{\text{m}}$ , $a_{11}^{\text{e}}$ , $a_{1-1}^{\text{e}}$

The actual solution to the inverse problem starts from random particle parameters as network input, for which we predict the spectral dependencies of the Mie angles with the trained ANN. From that output, we compute an objective function *f*, the Mean Absolute Error (MAE) of the ANN output and the target Mie angles at the desired wavelength, *λ*_d_, as

(2) $$\begin{array}{ll} f\left(\theta_{\text{target}},\theta_{\text{NN}};\lambda_{\text{d}}\right) & =\frac{1}{2}\underset{i\in \left\{E1,M1\right\}}{\sum }\left|\theta_{i,target}\left(\lambda_{\text{d}}\right),-\theta_{i,NN}\left(\lambda_{\text{d}}\right)\right|. \end{array}$$

Subsequently, the gradients of *f* with respect to the particle parameters $\frac{\partial f}{\partial \left(r,n\right)}$ are computed, and they are adjusted iteratively to minimize the MAE. This procedure is repeated for several initial parameters until we find a core–shell particle that provides the target Mie angles with high accuracy.

Unfortunately, the discretization of the refractive index values naturally leads to a restriction of the possible design space. Thus, the design Mie angles and those of the optimized stricture do not match in a perfect sense after that procedure, i.e., we found an agreement only up to two digits after the comma. However, the BIC is very sensitive to slight changes in the Mie angles. Hence, we perform a second optimization using the actual analytical computation of the Mie angles and varied the refractive index of the involved materials slightly for fine-tuning. This approach alone is significantly slower than ANN-assisted design, especially for several initial trials. Fortunately, in this work, the designed particle can be fine-tuned to achieve the required accuracy (eight digits after the comma) and grant the appearance of the BIC, given the results of the first ANN-assisted optimization approach as a single starting point. The fine-tuning of the refractive index of the shells could be carefully done by suitable doping of the respective material, which should be within reach with existing technology [48–50]. In summary, this procedure provides a general scheme implemented, herein, by a physical core–shell spherical particle [51–53] that can be used to form a metasurface that offers the BIC at the predefined frequency and incidence angle. Furthermore, the Mie angle approach, as presented, herein, can be expanded with the proper modifications for the case of core–shell dielectric disks [54].

### 2.3 Step 3: Application of the design methodology

The procedure presented above will now be applied to propose a metasurface with a square unit cell decorated by core–shell dielectric spheres that realize a BIC for a predefined lattice, frequency, and incidence angle. For this purpose, let us begin with the theoretical setup depicted in Figure 2 with the resulting Mie coefficients from Eq. (1), *a*_1_ = −0.4046 + 0.4908*i* and *b*_1_ = −0.5508 + 0.4974*i* for ${\tilde{\omega }}_{d}=0.517$ and *θ*_d_ = 55°. Afterward, the algorithm presented in the previous section will be utilized to design a realistic core–shell spherical particle that provides the calculated Mie coefficients *a*_1_ and *b*_1_. We define the dimension of the square lattice as *d* = 450 nm, thus, the desired operational wavelength is *λ*_d_ = 870 nm. The obtained set of parameters of the core–shell particle, i.e., the refractive indices and the geometric dimensions of the shells and core, of which the square array should consist, is shown in Figure 3(a). Next, we perform a multipolar decomposition of the scattered field [55] from the designed spherical particle upon illumination with a linearly polarized plane wave. As depicted in Figure 3(b), it possesses predominantly a dipolar response around the operational wavelength, *λ*_d_. Moreover, the Mie angles of the designed core–shell particle are calculated [45] and presented for the wavelength spectrum 500–1200 nm in Figure 3(c). Although the Mie angles are dispersive within the considered spectrum, they possess at the ${\tilde{\omega }}_{d}$ the required values for the designed operation, equal those requested by the theoretical model.

**Figure 4:**
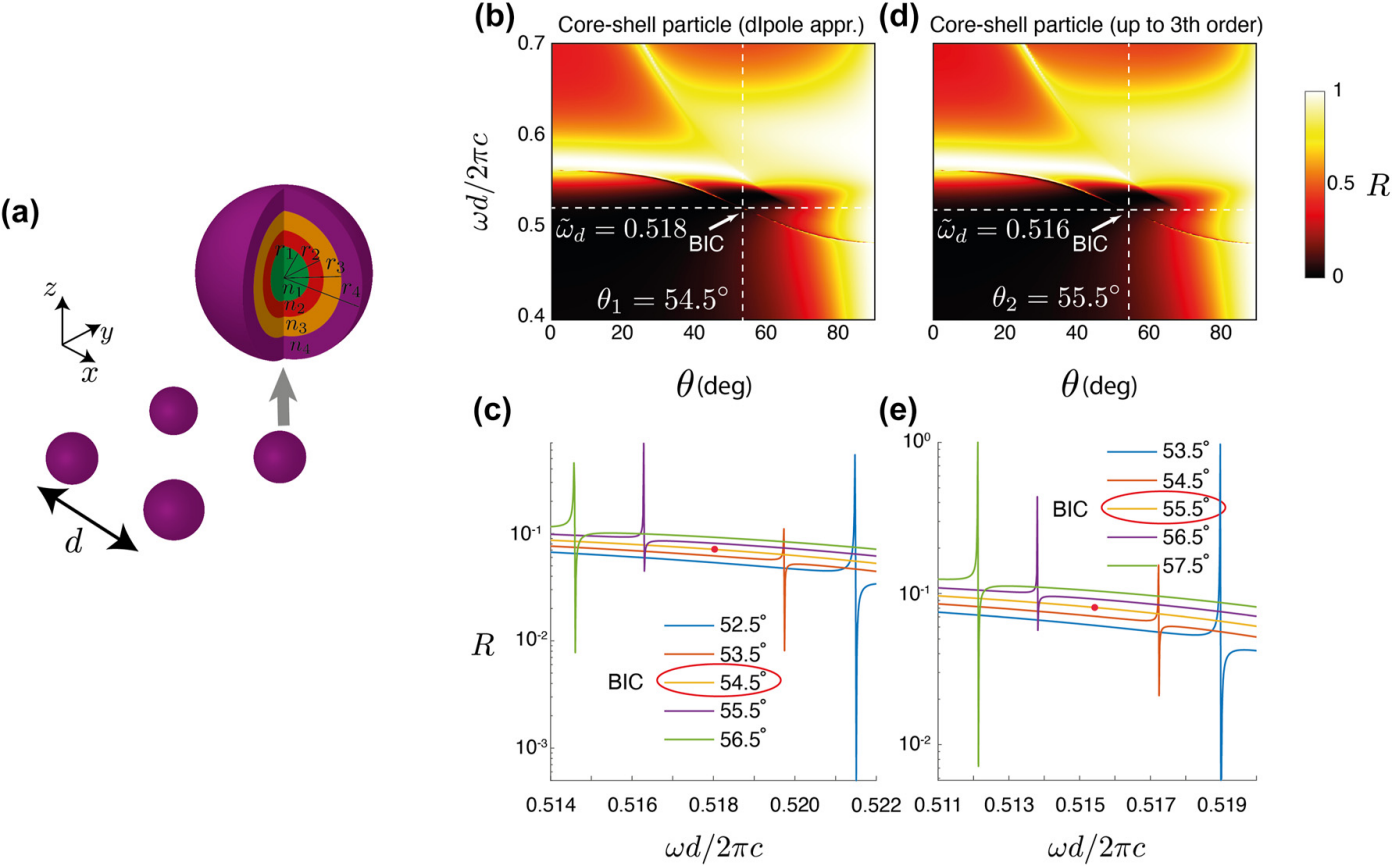
Designed metasurface: (a) The square lattice of core–shell particles. (b, d) Reflection coefficients of the actual metasurface as calculated with a full-wave Maxwell solver that exploits the T-matrix formalism. The reflection is shown as a function of the frequency and the incidence angle for a lattice dimension *d* = 450 nm. (b) Calculation in dipole approximation. (d) Calculation in octupole approximation. (c, e) The reflection coefficient versus the normalized frequency in proximity to the off-Γ BIC for a lattice dimension *d* and using the dipole or the octupole approximation, respectively.

Finally, the designed core–shell spherical particle is placed on a 2D square array (Figure 4(a)) and the optical response from the metasurface is analyzed with a dedicated T-matrix-based full-wave solver (see Supplementary Material) [42, 43]. In Figure 4(b)–(d), the reflection depending on the normalized frequency, *ωd*/2π*c*, and the incidence angle, *θ*, are shown when only dipole (first order) and up to octupole (third order) multipoles are considered in the response calculation, respectively [43]. Please note that the dipolar approximation would correspond to the assumption in the design process. However, the actual particle does not, of course, have a purely dipolar response, but also small, yet non-negligible, higher-order multipolar coefficients. These higher-order multipolar contributions usually need to be considered when the response from an actual metasurface is predicted.

Specifically, when only the dipolar response of the particle is considered (Figure 4(b)), a BIC is observed at the $\left(\tilde{\omega },\theta \right)=\left(0.518,54.5{}^{\circ}\right)$ , almost exactly on the desired location in the parameter space. A more clear view of the reported BIC point is given in Figure 4(c), where the reflection coefficient of the corresponding metasurface is plotted versus the normalized frequency for various angles of incidence. Thus, the deviation of the obtained core–shell particle metasurface from the desired BIC point in the theoretical dipole analysis is 1 %. The discrepancy can easily be explained by the fact that the realistic structure does not provide exactly the desired Mie coefficients. In other words, the consideration of actual materials causes these small deviations. Finally, to verify that the proposed design still fulfills the set goals when higher order multipoles are taken into account, the reflection coefficient from the core–shell particle metasurface is calculated when considering multipoles up to octupolar order, i.e., *j* = 3 [42, 43]. One can observe in Figure 4(d) and (e) that the resulting BIC position at $\left(\tilde{\omega },\theta \right)=\left(0.516,55.5{}^{\circ}\right)$ does not differ much from the desired one. Therefore, it can be safely deduced that the performance of the designed metasurface will remain the same in realistic conditions.

## 3 Discussion

The sensitivity of the BIC against deviations in the Mie angles parameterizing the optical response of the scatterers will now be discussed. This step is important for certifying the stability of the proposed algorithm. In most of the previous – mainly theoretical – work on metasurface inverse design, the robustness of the proposed design to deviations of material or geometrical parameters is not guaranteed as most of the ANNs work as a black box. In this paper, we suggested a more transparent scheme, where the problem is initially solved semi-analytically via the dipole approximation and, afterward, an artificial particle with the required polarizabilities is designed with the ANN.

**Figure 5:**
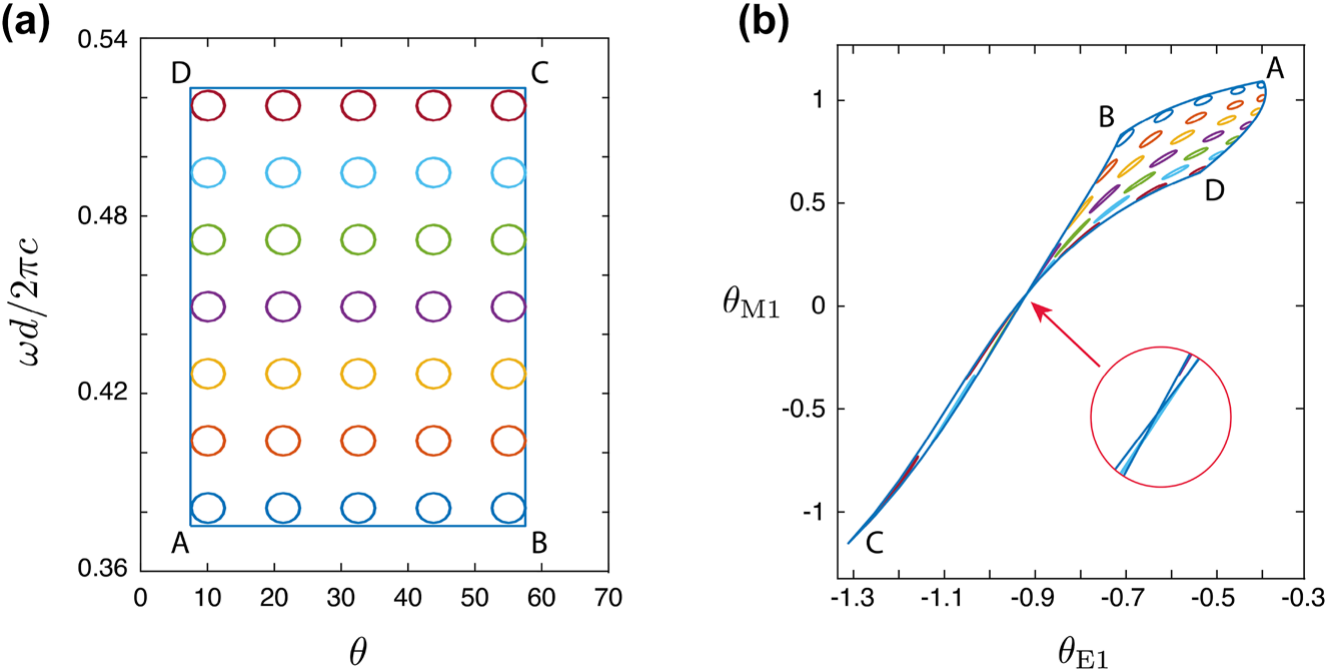
Robustness of the BICs. (a) The positions of the BICs for different angles *θ* (deg.) and normalized frequency $\tilde{\omega }$ values: circles and contour in the form of a rectangle *ABCD*. (b) The values of the Mie angles for BICs from panel a.

Figure 5 demonstrates how the position of the BIC for different angles *θ* and frequency $\tilde{\omega }$ values translates to values of the required Mie angles: each point in Figure 5(a) corresponds to a pair of Mie angles (*θ*_M1_ and *θ*_E1_), which characterize the magnetic and electric dipole response of the single scatterer, which is necessary for the existence of BIC. The bounding box as a rectangle *ABCD* is transformed very strongly in the plane of the Mie angles. That can also be seen in the individual circles that provide some insights into the robustness. Generally, for a robust BIC, we require each area in the plane of the Mie angles to be as large as possible and as circular as possible. That would suggest that a given Mie coefficient only needs to be roughly hit to ensure that a BIC appears in the target area given by the circles in the plane spanned by the frequency and incident angle. However, the BIC is extremely sensitive for a strongly reduced area of the surface in the plane of the Mie angles. Thus, for that case, the Mie angles must be precisely met to cause a BIC at predefined frequencies and angles. Moreover, we notice that some circles are stretched and elongated in the plane of the Mie angles. Here, we will speak about conditional robustness. In this case, many Mie angles, for, let’s say, the electric dipole moment, permit the observation of the BIC in the target range of parameters. However, the other Mie angle must be carefully tuned to be within the stretched circle. One can roughly conclude that the most robust region is in the zone of lower frequencies and shorter angles (close to the corner *A*). However, the excitations of BICs in the related parameter regime are difficult for practical systems. The sizes of realistic spheres, obtained as a result of the deep learning-assisted engineering step, compare very well to the period of the metasurface. Therefore, the BIC position could shift a lot relative to the desired angles and frequencies since the renormalization is substantial in that parameter domain.

In Figure 5(b), the intersection of two lines of the bounding box occurs as a further interesting phenomenon. It means that for these Mie angles at the point of intersection, the metasurface sustains two distinct BICs for different pairs of frequency and incidence angles. This case is discussed in more detail in the Supplementary Material.

Although, in this work, we have utilized the dipole approximation to design the meta-atoms, i.e., only up to the first multipolar order, the core idea behind the presented methodology is general and can be applied to higher-order multipoles or more complex scatterers. Provided that the general eigenvalue problem (see (6) in METHODS) can be solved under some approximations and the solutions corresponding to the BIC resonances can be recognized, the Mie angles that lead to the desired BIC can be expressed. For example, in [43], for a square metasurface illuminated by a normally-incident plane wave and consisting of an isotropic particle with only a magnetic dipole and an electric quadrupole excited, a BIC point was identified, or the required (*b*_1_, *a*_2_) values. Then, a suitable inverse design technique can be used to identify an actual meta-atom that offers these desired Mie angles. In the work at hand, the acquired Mie coefficients were assigned to a core–shell particle via the deep learning algorithm. These considerations can be extended to more complex, e.g., anisotropic or bi-anisotropic scatterers and can accommodate an increasing number of multipolar orders. Approximate models for the T matrix could be utilized for that purpose [56, 57] or more advanced neural networks can be trained [58].

Moreover, the proposed scheme could be expanded within dipole approximation, in order to include material dispersion. If the dipole Mie coefficients are modeled for a Lorentzian dispersion [45], one can employ an optimization technique, to obtain a set of a Lorentzian resonant frequency and radiative losses (see Section 3 in Supplementary Material) that will provide a BIC via a metasurface for a given angle of incidence and operational frequency and, finally, try to associate the results with practical dispersive materials.

Furthermore, although “true” BICs with an infinite *Q* cannot exist in structures that not extend to infinity for at least one dimension [1], an expansion of the proposed procedure can be applied for finite arrays or non-periodic metasurfaces to design quasi-BIC resonances for a desired set (*θ*_inc_, *ωd*/*λ*), with a very high, yet finite *Q* [59]. Specifically, in finite structures, where the unit cells are not equivalent to each other, one could model the problem within dipole approximation, afterward, find the quasi-BIC conditions via the eigenvalues of the system and, finally, acquire the individual design of each consisting particle with the use of an optimization scheme.

Lastly, the same procedure as above could also be applied to metasurfaces with other functionalities, such as lenses or arbitrary holograms, where different types or shapes of scatterers form the final 2D array. Initially one has to acquire the scattering properties of each unit cell via Mie angles. Again, the unit-cells of the metasurface are different to each other, thus, solving the more complicated eigenvalue problem via the dipole approximation will, now, provide the quasi-BIC conditions.

In conclusion, the generalized design scheme presented, herein, for infinite, periodic structures composed of spherical, isotropic particles can be extrapolated to finite 2D arrays or 2D arrays with non-identical particles. The only difference would be the need to solve multiple-particle problems and parametrize each unit-cell with a required number of Mie angles. Nevertheless, the main idea presented in this work remains the same.

## 4 Conclusions

In summary, the physics-inspired inverse design procedure for all-dielectric metasurfaces sustaining an accidental BIC at a certain frequency and angle of incidence was developed in this work. The methodology utilized a smart combination of a semi-analytical approach and a dedicated machine-learning algorithm. Firstly, the problem was solved within a theoretical model considering all-dielectric meta-atoms using the dipole approximation and the T-matrix technique. The existence condition for an accidental BIC was derived in terms of Mie angles. Secondly, using a dedicated artificial neural network, the geometrical and material parameters of a core–shell, spherical particle that offered the desired optical response provided by the BIC condition were identified. In this way, a physically feasible meta-atom was found that provided the desired BIC when placed on an infinite 2D array. Finally, the design was numerically verified, and the robustness of the designed position BIC in *k*-space was tested by considering higher-order multipoles.

## 5 Methods

### 5.1 The T-matrix formulation for 2D arrays and BIC identification

Considering a metasurface composed of a square array of isotropic and non-absorbing particles, as shown in Figure 2(a). The surrounding medium is a vacuum.

If an arbitrary scatterer is placed in an infinite homogeneous background and the vector spherical harmonics (VSH) basis is employed to expand the fields, the scattering response to an incident electromagnetic wave can be described using the T matrix, or ${\bar{\bar{T}}}_{0}$ , as

(3) $$\left[\begin{array}{c} a^{\text{e}}\\ a^{m} \end{array}\right]={\bar{\bar{T}}}_{0}\left[\begin{array}{c} q^{\text{e}}\\ q^{m} \end{array}\right]=\left[\begin{array}{cc} {\bar{\bar{T}}}^{\;ee} & {\bar{\bar{T}}}^{\;em}\\ {\bar{\bar{T}}}^{\;me} & {\bar{\bar{T}}}^{\;mm} \end{array}\right]\left[\begin{array}{c} q^{\text{e}}\\ q^{m} \end{array}\right].$$

Vectors **q** ^{e,*m*}^ contain the incident wave electric or magnetic coefficients, respectively, while **a** ^{e,*m*}^ contain the scattering electric or magnetic coefficients, respectively [60]. For each multipolar expansion order j∈N , the size of the aforementioned vectors increases by 2*j* + 1. Prior knowledge of the T matrix can predict the electromagnetic response of a scatterer since it depends on the scatterer’s geometry, constituting materials, and surrounding material. The elements of the T matrix can generally be extracted up to a preset expansion order via numerical simulations [55, 61], while for special geometries, such as spheres, cylinders, etc., the T matrix elements can be calculated analytically. The particular case of isotropic particles, i.e., spherical ones, is very interesting because apart from enabling analytic calculations, the respective T matrices become diagonal, or, ${\bar{\bar{T}}}^{\;em}={\bar{\bar{T}}}^{\;me}=\bar{\bar{0}}$ and $T_{il}^{\;ee}=T_{il}^{\;mm}=0$ , for *i* ≠ *l*, with {*i*, *l*} ∈ [−*j*, *j*], while ${\bar{\bar{T}}}_{j}^{\;ee}$ and ${\bar{\bar{T}}}_{j}^{\;mm}$ become scalar matrices for each multipolar order *j*, or, ${\bar{\bar{T}}}_{j}^{\;ee}=-a_{j}\bar{\bar{I}}$ and ${\bar{\bar{T}}}_{j}^{\;mm}=-b_{j}\bar{\bar{I}}$ . The parameters *a*_*j*_ and *b*_*j*_ are called the *Mie coefficients* [62].

For an isotropic particle, it is convenient to parameterize the coefficients with *Mie angles* [45]. In particular, for lossless particles and when only dipoles are considered, i.e., *j* = 1, all possible Mie coefficients can be expressed using two parameters *θ*_E1_ and *θ*_M1_, or

(4a) $$a_{1}=\frac{1}{1-i\;\tan\theta_{E1}},\;\;-\frac{\pi }{2}\leq \theta_{E1}\leq \frac{\pi }{2},$$

(4b) $$b_{1}=\frac{1}{1-i\;\tan\theta_{M1}},\;\;-\frac{\pi }{2}\leq \theta_{M1}\leq \frac{\pi }{2}.$$

As a result, the optical response of the corresponding scatterer can be parameterized with the two Mie angles above, while still having a clear physical picture. This formulation is very important because the control parameters, namely *θ*_E1_ and *θ*_M1_, are placed between an upper and lower boundary, which will aid optimization methods and subsequent metasurface design. Furthermore, similar expressions involving Mie angles, like the ones of (4) above, exist for higher-order multipoles, the addition of absorption or Lorentzian dispersion for Mie coefficients (see Supplementary Material).

Let us now assume an infinite square array composed of arbitrary, identical, absorption-less, spherical particles placed in a homogeneous material, as depicted in Figure 1. The response of the metasurface can be calculated via the T matrix of the particle by employing microscopic models [42, 43, 63, 64]. An important aspect to be noted herein is that the T matrix of the particle within the metasurface is *re-normalized* due to the particle interaction. Thus, in 2D array models, the isolated particle’s T matrix, ${\bar{\bar{T}}}_{0}$ , from (3) is replaced with the *effective T matrix* calculated via [63]

(5) $${\bar{\bar{T}}}_{\text{eff}}={\left(\bar{\bar{I}}-{\bar{\bar{T}}}_{0}{\bar{\bar{C}}}_{s}\right)}^{-1}{\bar{\bar{T}}}_{0},$$

where $\bar{\bar{I}}$ is the identity matrix, and ${\bar{\bar{C}}}_{s}$ is the *lattice coupling matrix* expressed in spherical coordinates, which is a function of the unit cell dimension, the frequency, and the wavevector, and is calculated, herein, via rapidly converging summations using Ewald’s method [42, 44, 65]. The analytical method based on a multipolar expansion that is used in this work for the calculation of the response of an infinite 2D square array [43] is elaborated in the Supplementary Material.

It should be stressed that, although the scattering response of a single particle and of the subsequent 2D array is provided here using the spherical coordinates and the T matrix approach, the analysis that will follow is valid and interchangeable with the use of multipoles in Cartesian coordinates and the polarizability matrix. In particular, one can obtain the T matrix of a particle from the polarizability matrix, and vice versa using the appropriate transformation matrices for each multipolar order [43, 66] (Table 2). Additionally, modifications of the same matrices can transform multipoles of the same type and order from a spherical to a Cartesian basis and vice versa, as presented in the Supplementary Materials for multipoles up to the *j* = 3 order.

After defining the multipolar description of the electromagnetic response of 2D arrays composed of isotropic particles, next, we will present the procedure for identifying the presence of BICs after setting certain goals. First, let us theoretically acquire the BIC position using the multipolar expansion technique [43, 67–69]. If we again assume a square array composed of identical and isotropic particles, its response to an incident field can be described by substituting (3) and (5) into the equations that describe the response of a 2D array [42, 43] (see Supplementary Material), and, thus, a linear system of equations is formed. Solving the eigenvalue problem leads to the modes of the array, including, in this case, the trapped ones that do not couple with radiation channels, i.e., the BICs. Therefore, if we invert the square matrix of the system to the left side of (3) and set the excitation to zero, or **q** ^{e,m}^ = **0**, the resulting homogeneous system will have a non-trivial solution if the determinant of the matrix is zero [26, 27, 43]. In particular, after some algebra, the BIC condition is reduced to

(6) $$\left|\;\bar{\bar{I}}-{\bar{\bar{T}}}_{0}\;{\bar{\bar{C}}}_{s}\;\right|=0.$$

The symmetry of the resonators is tightly related to their mode structure and multipole content, which determine the linear and non-linear response of the resonator. Using group theory, it is possible to classify the eigenmodes into irreducible representations and understand their multipole content [70–72]. Our model is characterized only by the dipole response. Table 1 shows how the dipole moments correspond to different types of eigenmodes for a square array of particles outside the off-Γ point in a spherical basis. The system is characterized by the *C*_2*v*_ symmetry group in direction Γ*X*. There are only 4 types of modes: *A*_1_, *A*_2_, *B*_1_, and *B*_2_. Destructive interference of the eigenmodes of one type is necessary for BICs formed by the Friedrich-Wintgen mechanism. It can be concluded that in this model, parametric BIC exists only in modes of types *B*_1_ and *B*_2_. The expression (6) is general in nature and can be used for any type of lattice or particle in a homogenous medium [43]. Due to its complexity, (6) can only be solved numerically in its general form, i.e., for higher-order multipoles or more diverse lattices. Nevertheless, for specific reduced cases, versatile analytic solutions can be found, as demonstrated in [43] for the case of the coupled electric dipole – magnetic quadrupole on a square lattice and a normal, TM wave incidence. In this work, we consider a square lattice decorated by isotropic and lossless particles, whose response is expanded only up to dipolar order, or *j* = 1. Then, because the elements of the lattice interaction matrix, ${\bar{\bar{C}}}_{s}$ , can be pre-calculated for a specific incident wavevector, **k** ^inc^, and a normalized lattice dimension, *d*/*λ*, eventually, (6) can be solved for *j* = 1 with the Mie angles of (4) as the unknowns.

## Supplementary Material

Supplementary Material Details
