# Supplementary material for: Inverse design of all-dielectric metasurfaces with accidental bound states in the continuum
Source: Nanophotonics. 2023 Sep 22;12(19):3767–79. doi: 10.1515/nanoph-2023-0373 (PMC11635925; doi:10.1515/nanoph-2023-0373)
Supplement: Supplementary file 1 — Supplementary Material Details [file j_nanoph-2023-0373_suppl_001.pdf]

# Supplementary Material for "Inverse Design of All-dielectric Metasurfaces with Accidental Bound States in the Continuum"

Sergei Gladyshev*,†, Theodosios D. Karamanos*,†, Lina Kuhn, Dominik Beutel, Thomas Weiss, Carsten Rockstuhl, Andrey Bogdanov

*e-mail: sergei.gladyshev@uni-graz.at, theodosios.karamanos@espci.fr.
†These authors have contributed equally.

## 1 Field expansion via vector spherical harmonics

Assume a particle positioned inside an infinite, non-dispersive, linear, homogeneous, and isotropic medium with an electromagnetic field illuminating it. The total electric field in the spatial domain outside and around the particle at an angular frequency $\omega$ consists of the incident and scattered fields. Each of these fields can be expanded using vector spherical harmonics (VSHs) [1] as,

$$\mathbf{E}_{\mathrm{inc}}(\mathbf{r}) = \sum_{j=1}^{\infty} \sum_{m=-j}^{j} q^{\mathrm{e}}_{jm} \mathbf{N}^{(1)}_{jm}(k\mathbf{r}) + q^{\mathrm{m}}_{jm} \mathbf{M}^{(1)}_{jm}(k\mathbf{r}), \tag{S1a}$$

$$\mathbf{E}_{\mathrm{sc}}(\mathbf{r}) = \sum_{j=1}^{\infty} \sum_{m=-j}^{j} a^{\mathrm{e}}_{jm} \mathbf{N}^{(3)}_{jm}(k\mathbf{r}) + a^{\mathrm{m}}_{jm} \mathbf{M}^{(3)}_{jm}(k\mathbf{r}), \tag{S1b}$$

with $q^{v}_{jm}$ and $a^{v}_{jm}$, $j = \{1, 2, 3...\}$ - positive interger, $m = \{-j, ..., j\}$, $v = \{\mathrm{e}, \mathrm{m}\}$, the electric/magnetic incident and scattered field expansion coefficients, respectively, or simply the incident and scattering coefficients. The wavenumber $k$ corresponds to the medium that surrounds the particle, and $r > r_c$, with $r_c$ being the radius of the smallest sphere that circumscribes the particle. Moreover, the VSH are defined as,

$$\mathbf{M}^{(l)}_{jm} = \gamma_{jm} \nabla \times \left[ \hat{\mathbf{r}} z^{(l)}_{j}(kr) P^{m}_{j}(\cos\theta) e^{\mathrm{i}m\phi} \right], \tag{S2a}$$

$$\mathbf{N}^{(l)}_{jm} = \frac{1}{k} \nabla \times \mathbf{M}^{(l)}_{jm}, \tag{S2b}$$

with

$$\gamma_{jm} = \sqrt{\frac{(2j+1)}{4\pi j(j+1)}} \sqrt{\frac{(j-m)!}{(j+m)!}}, \tag{S2c}$$

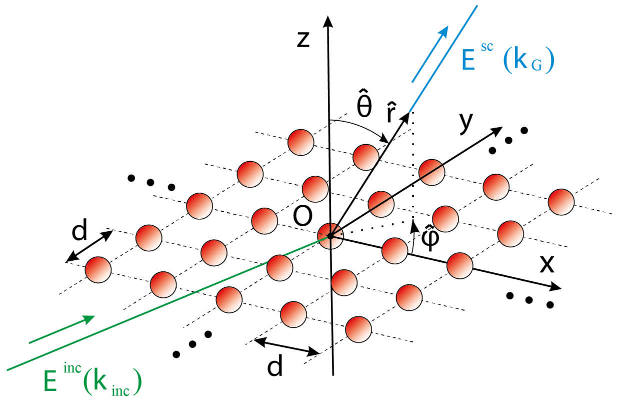


Figure S1: A square lattice of identical particles along with the set-up Cartesian and spherical coordinate systems. The 2D array is illuminated by an incident wave, $\mathbf{E}^{\mathrm{inc}}$, and subsequently emits a scattered wave, $\mathbf{E}^{\mathrm{sc}}$.

with $l = 1$ for the incident field and $l = 3$ for the scattered one. Additionally, $z_j^{(1)}(x) = j_j(x)$ is the spherical Bessel function of the first kind, while $z_j^{(3)}(x) = h_j^{(1)}(x)$ is the spherical Hankel function of the first kind. Finally, $P_j^m(x)$ is the associated Legendre polynomial.

## 2 Scattering from 2D arrays via multipolar expansion

Let us assume an infinite square array composed of arbitrary, identical and absorption-less particles, as displayed in Fig. S1, placed in a homogeneous material with $\varepsilon_{\mathrm{r}}$ and $\mu_{\mathrm{r}}$ being the relative permittivity and permeability of the medium, respectively. Let us consider, also, an electromagnetic plane wave impinging onto this 2D array, with an electric field, $\mathbf{E}^{\mathrm{inc}} = \mathbf{E}_0\, e^{\mathrm{i}\mathbf{k}^{\mathrm{inc}}\cdot\mathbf{r}}$, with a wavevector, $\mathbf{k}^{\mathrm{inc}} = k_x^{\mathrm{inc}}\,\hat{\mathbf{x}} + k_y^{\mathrm{inc}}\,\hat{\mathbf{y}} + k_z^{\mathrm{inc}}\,\hat{\mathbf{z}}$, and a magnitude, $E_0 = |\mathbf{E}_0|$. After employing the VSH formulation and the multipolar expansion method, the scattered fields from each diffraction order, defined by the reciprocal lattice $\mathbf{G}$, can be expressed as [2, 3, 4]

$$\mathbf{E}^{\mathrm{sc}} = \frac{\mathrm{i}\sqrt{\pi}}{2d^2k^2}\,\frac{e^{\mathrm{i}\mathbf{k}_{\mathbf{G}}^{\pm}\cdot\mathbf{r}}}{|\cos\theta|}\sum_j \frac{\sqrt{2j+1}}{\mathrm{i}^j}\begin{bmatrix} \mathbf{W}_j & \mathbf{W}'_j \\ \mathrm{i}\mathbf{W}'_j & \mathrm{i}\mathbf{W}_j \end{bmatrix}\begin{bmatrix} \mathbf{a}_j^{\mathrm{e}} \\ \mathbf{a}_j^{\mathrm{m}} \end{bmatrix}, \tag{S3}$$

where $d$ is the period of the square lattice, $\mathbf{k}_{\mathbf{G}}^{\pm}$ is the wavevector of the respective mode $\mathbf{G}$, $\theta$ is the polar angle of the wavevector and the $\pm$ signs refer to transmission or reflection, respectively. The wavevector $\mathbf{k}_{\mathbf{G}}^{\pm}$ for a square lattice and each of diffraction orders are calculated as [2]

$$\mathbf{k}_{\mathbf{G}}^{\pm} = k_{\mathbf{G},x}\,\hat{\mathbf{x}} + k_{\mathbf{G},y}\,\hat{\mathbf{y}} + k_{\mathbf{G},z}^{\pm}\,\hat{\mathbf{z}}, \tag{S4a}$$

$$k_{\mathbf{G},x} = k_x^{\mathrm{inc}} + \frac{2\pi n_1}{d}, \quad k_{\mathbf{G},y} = k_y^{\mathrm{inc}} + \frac{2\pi n_2}{d}, \tag{S4b}$$

$$k_{\mathbf{G},z}^{\pm} = \pm\sqrt{k^2 - \left(k_x^{\mathrm{inc}} + \frac{2\pi n_1}{d}\right)^2 - \left(k_y^{\mathrm{inc}} + \frac{2\pi n_2}{d}\right)^2} = k\cos\theta, \tag{S4c}$$

where $n_1, n_2 \in \mathbb{Z}$ are the diffraction orders. The respective elements of the vectors $\mathbf{W}_j$ and $\mathbf{W}'_j$ are $W_{jm} = \frac{m}{\cos(\theta)} P_j^m(\cos\theta)$ and $W'_{jm} = \frac{\partial}{\partial\theta} P_j^m(\cos\theta)$, with $P_j^m(x)$ being the associated Legendre polynomial. The vectors $\mathbf{a}_j^{\{\mathrm{e,m}\}}$ in (S3) are the effective electric/magnetic scattering coefficients of the particle inside the lattice, for each multipolar order $j$, and they are calculated via the effective T matrix, i.e. the renormalized T matrix of the constituting particle within the lattice, as presented in (3) of the Main article.

# 3 Mie angles

Considering an isotropic particle, namely a sphere, made from an absorbing material, its Mie coefficients can be expressed as[5]

$$a_j = \frac{1}{1 - \mathrm{i}\tan\theta_{\mathrm{E}j} + \tan\theta'_{\mathrm{E}j}}, \qquad -\frac{\pi}{2} \le \theta_{\mathrm{E}j} \le \frac{\pi}{2},\ \ 0 \le \theta'_{\mathrm{E}j} \le \frac{\pi}{2}, \tag{S5a}$$

$$b_j = \frac{1}{1 - \mathrm{i}\tan\theta_{\mathrm{M}j} + \tan\theta'_{\mathrm{M}j}}, \qquad -\frac{\pi}{2} \le \theta_{\mathrm{M}j} \le \frac{\pi}{2},\ \ 0 \le \theta'_{\mathrm{E}j} \le \frac{\pi}{2}. \tag{S5b}$$

where $\theta_{\mathrm{E}j}$ and $\theta_{\mathrm{M}j}$ are the *detuning Mie angles*, while $\theta'_{\mathrm{E}j}$ and $\theta'_{\mathrm{M}j}$ are the *absorption Mie angles*.

Moreover, if, e.g., the electric dipole Mie coefficient is modelled using a Lorentzian dispersion , then it can be expressed as a function of the respective Mie angle, or [5],

$$a_1 = \frac{\mathrm{i}\gamma_r^e/2}{(\omega - \omega_{0e}) + \mathrm{i}(\gamma_{nr}^e + \gamma_r^e)/2}, \quad \text{with } \tan\theta_{\mathrm{E1}} = \frac{2(\omega - \omega_{0e})}{\gamma_r^e} \text{ and } \tan\theta'_{\mathrm{E1}} = \frac{\gamma_{nr}^e}{\gamma_r^e}, \tag{S6}$$

where $\omega_{0e}$ being the resonant frequency and $\gamma_r^e$ and $\gamma_{nr}^e$, the radiative and non-radiative (absorption) losses, respectively, for the specific electric dipole. Other Mie coefficients either magnetic or of higher order can be modelled in a similar fashion with Lorentz dispersion using Mie angles [5]. Therefore, the Mie angle formulation can also be useful for the study and design of particles while taking into account a frequency-dependent scattering response.

Let us now examine a BIC example considering Mie coefficients with Lorentz dispersion. If we consider an isotropic particle represented by the Mie coefficients $a_1$ and $b_1$, placed on a cubic lattice with dimension $d$, we can find the BIC point by using (S3). In particular, assuming no absorption and a non-dispersive radiative losses, one can sweep the resonant frequencies $\omega_{0e}$ and $\omega_{0m}$, for specific operational normalized frequency, $\tilde{\omega}_d = \omega\, d/2\pi c$, and angle of incidence, $\theta_d$, as goals, and the resulting reflection coefficient from the corresponding 2D array is depicted in Fig. S2. A BIC is observed for the resonant frequencies $(\omega_{0e}, \omega_{0m}) \cdot d/2\pi c = (0.75, 0.55)$, electric and magnetic, respectively, for $\omega d/2\pi c = 0.55$ and $\theta_d = 35^{\mathrm{o}}$. Thus, one can, afterward, engineer a particle with the specific parameters using an optimization method to realize this specific BIC behavior with metasurfaces.

For the case of no losses, the absorption Mie angles turn to $\theta'_{\mathrm{E}j} = \theta'_{\mathrm{M}j} = 0$ and (S5) become the Mie angle expressions for *lossless particles* [5], as applied in the Main article. In particular, when a scatterer is isotropic and without absorption, the respective Mie coefficients can be described in terms of

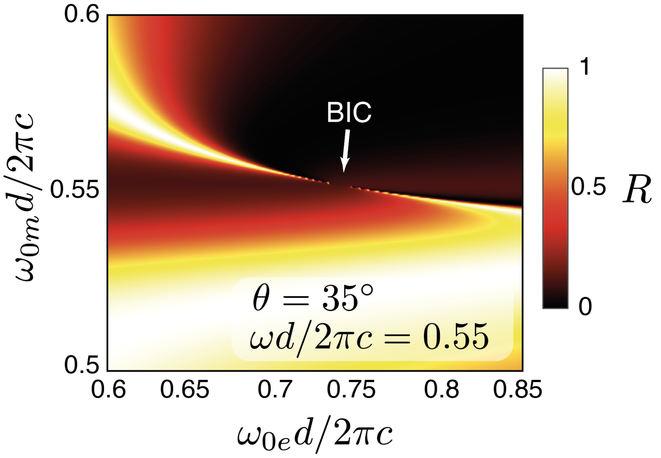


Figure S2: Reflection coefficient magnitude from a cubic 2D array with identical $a_1$ and $b_1$ dipoles exhibiting Lorentz dispersion, as demonstrated in (S6), versus the normalized electric and magnetic resonant frequencies. The scatterers are assumed to be lossless ($\gamma_{nr} = 0$, while the radiative losses are, $\gamma_r^e = 1100c/d$, $\gamma_r^m = 360c/d$, the angle of incidence, $\theta_{\rm inc} = 35^o$, and the normalized frequency, $\omega d/2\pi c = 0.55$.

*Mie angles* as

$$a_j = \frac{1}{1 - \mathrm{i}\tan\theta_{\mathrm{E}j}}, \; -\frac{\pi}{2} \leq \theta_{\mathrm{E}j} \leq \frac{\pi}{2}, \tag{S7a}$$

$$b_j = \frac{1}{1 - \mathrm{i}\tan\theta_{\mathrm{M}j}}, \; -\frac{\pi}{2} \leq \theta_{\mathrm{M}j} \leq \frac{\pi}{2}. \tag{S7b}$$

The Mie angle formulation enables accessing all possible values of the Mie coefficients for an existing isotropic and without absorption particle, simply by sweeping through all possible angles, $\theta_{\mathrm{E}j}$ and $\theta_{\mathrm{M}j}$, as declared in (S7). This versatile formulation can, subsequently, aid the investigation of optical phenomena and design of novel devices [6, 4], and is further applied in the Main article.

# 4 From Cartesian to Spherical multipoles

Although the scattering response of a single particle and of the subsequent 2D array is provided in this work using the spherical coordinates and the T matrix approach, the proposed analysis is valid and interchangeable with the use of multipoles in cartesian coordinates and the polarizability matrix. In particular, one can obtain the T matrix of a particle from the polarizability matrix and vice versa using the appropriate transformation matrices for each multipolar order [7, 4]. Additionally, modifications of the same matrices can transform multipoles of the same type and order from a spherical to a cartesian basis and vice versa. For example, in the case of electric dipoles, the transformation between cartesian

and spherical coordinates is performed as [7],

$$\begin{bmatrix} p_x \\ p_y \\ p_z \end{bmatrix} = \frac{\varepsilon\sqrt{3\pi}}{\mathrm{i}k^3} \begin{bmatrix} 1 & 0 & -1 \\ -\mathrm{i} & 0 & -\mathrm{i} \\ 0 & \sqrt{2} & 0 \end{bmatrix} \begin{bmatrix} a^{\mathrm{e}}_{1-1} \\ a^{\mathrm{e}}_{10} \\ a^{\mathrm{e}}_{11} \end{bmatrix}, \tag{S8a}$$

$$\begin{bmatrix} m_x \\ m_y \\ m_z \end{bmatrix} = -\frac{\sqrt{3\pi}}{\eta k^3} \begin{bmatrix} 1 & 0 & -1 \\ -\mathrm{i} & 0 & -\mathrm{i} \\ 0 & \sqrt{2} & 0 \end{bmatrix} \begin{bmatrix} a^{\mathrm{m}}_{1-1} \\ a^{\mathrm{m}}_{10} \\ a^{\mathrm{m}}_{11} \end{bmatrix}. \tag{S8b}$$

Similarly, the transformation above can be performed for quadrupoles, or $j = 2$, as [7, 4]

$$\begin{bmatrix} Q^{\mathrm{e}}_{xy} \\ Q^{\mathrm{e}}_{yz} \\ \frac{\sqrt{3}}{2} Q^{\mathrm{e}}_{zz} \\ Q^{\mathrm{e}}_{xz} \\ \frac{1}{2}\big(Q^{\mathrm{e}}_{xx} - Q^{\mathrm{e}}_{yy}\big) \end{bmatrix} = \frac{\varepsilon\, 6\sqrt{5\pi}}{\mathrm{i}k^4} \begin{bmatrix} -\mathrm{i} & 0 & 0 & 0 & \mathrm{i} \\ 0 & -\mathrm{i} & 0 & -\mathrm{i} & 0 \\ 0 & 0 & \sqrt{2} & 0 & 0 \\ 0 & 1 & 0 & -1 & 0 \\ 1 & 0 & 0 & 0 & 1 \end{bmatrix} \begin{bmatrix} a^{\mathrm{e}}_{2-2} \\ a^{\mathrm{e}}_{2-1} \\ a^{\mathrm{e}}_{20} \\ a^{\mathrm{e}}_{21} \\ a^{\mathrm{e}}_{22} \end{bmatrix}, \tag{S9a}$$

$$\begin{bmatrix} Q^{\mathrm{m}}_{xy} \\ Q^{\mathrm{m}}_{yz} \\ \frac{\sqrt{3}}{2} Q^{\mathrm{m}}_{zz} \\ Q^{\mathrm{m}}_{xz} \\ \frac{1}{2}\big(Q^{\mathrm{m}}_{xx} - Q^{\mathrm{m}}_{yy}\big) \end{bmatrix} = -\frac{6\sqrt{5\pi}}{\eta k^4} \begin{bmatrix} -\mathrm{i} & 0 & 0 & 0 & \mathrm{i} \\ 0 & -\mathrm{i} & 0 & -\mathrm{i} & 0 \\ 0 & 0 & \sqrt{2} & 0 & 0 \\ 0 & 1 & 0 & -1 & 0 \\ 1 & 0 & 0 & 0 & 1 \end{bmatrix} \begin{bmatrix} a^{\mathrm{m}}_{2-2} \\ a^{\mathrm{m}}_{2-1} \\ a^{\mathrm{m}}_{20} \\ a^{\mathrm{m}}_{21} \\ a^{\mathrm{m}}_{22} \end{bmatrix}. \tag{S9b}$$

The vectors on the left side of (S9) represent one irreducible set of quadrupoles on Cartesian coordinates. The remaining quadrupoles can be calculated using symmetries, i.e. $Q_{ij} = Q_{ji}$ and $Q_{xx} + Q_{yy} + Q_{zz} = 0$. A relation between octupoles ($j = 3$) in Cartesian and spherical coordinates can additionally be found in [4]. Extending the transformations for higher orders is, afterward, becoming increasingly difficult and cumbersome, while the intuition provided by the Cartesian coordinates diminishes. Thus, for $j > 3$ the use of multipoles represented in spherical coordinates is advised.

When only dipoles are considered, beginning from (S8), the transformation between the polarizability and T matrices can be derived as [7, 4]

$$\bar{\bar{\alpha}}^{vv'} = -\frac{\mathrm{i}k^3}{6\pi} \begin{bmatrix} 1 & 0 & -1 \\ -\mathrm{i} & 0 & -\mathrm{i} \\ 0 & \sqrt{2} & 0 \end{bmatrix} \bar{\bar{T}}^{vv'} \begin{bmatrix} 1 & 0 & -1 \\ -\mathrm{i} & 0 & -\mathrm{i} \\ 0 & \sqrt{2} & 0 \end{bmatrix}^{-1}, \qquad \{v, v'\} = \{\mathrm{e}, \mathrm{m}\}. \tag{S10a}$$

with

$$\begin{bmatrix} \mathbf{p} \\ \mathrm{i}\eta\mathbf{m} \end{bmatrix} = \bar{\bar{\alpha}} \begin{bmatrix} \varepsilon\mathbf{E} \\ \mathrm{i}\eta\mathbf{H} \end{bmatrix} \tag{S10b}$$

with the dipolar polarizabilities defined as in [4]. Moreover, if spherical particles are assumed, as it is the case in this work, then, the total polarizability matrix becomes diagonal, or $\bar{\bar{\alpha}} = \mathrm{diag}\big\{\bar{\bar{\alpha}}^{\mathrm{ee}}\ \bar{\bar{\alpha}}^{\mathrm{mm}}\big\} = \mathrm{diag}\big\{\alpha^{\mathrm{ee}}_{xx}\ \alpha^{\mathrm{ee}}_{yy}\ \alpha^{\mathrm{ee}}_{zz}\ \alpha^{\mathrm{mm}}_{xx}\ \alpha^{\mathrm{mm}}_{yy}\ \alpha^{\mathrm{mm}}_{zz}\big\} = \mathrm{diag}\big\{\alpha^{\mathrm{e}}\ \alpha^{\mathrm{e}}\ \alpha^{\mathrm{e}}\ \alpha^{\mathrm{m}}\ \alpha^{\mathrm{m}}\ \alpha^{\mathrm{m}}\big\}$. Similarly, the T matrix becomes diagonal, or $\bar{\bar{T}} = \mathrm{diag}\big\{\bar{\bar{T}}^{\mathrm{ee}}\ \bar{\bar{T}}^{\mathrm{mm}}\big\} = \mathrm{diag}\big\{-a_1\ -a_1\ -a_1\ -b_1\ -b_1\ -b_1\big\}$, with $a_1$ and $b_1$ being the Mie coefficients, as defined above. Therefore, the polarizabilities of a spherical particle can be directly derived from the Mie coefficients through (S10) as

$$\alpha^{\mathrm{e}} = \frac{\mathrm{i}k^3}{6\pi}\, a_1 \qquad \text{and} \qquad \alpha^{\mathrm{m}} = \frac{\mathrm{i}k^3}{6\pi}\, b_1. \tag{S11}$$

# 5 BIC identification for a dipole metasurface

After having introduced the theoretical tools to describe the electromagnetic response of 2D arrays composed of isotropic particles, in this section, we will describe the procedure of identifying the presence of BICs after setting certain goals.

First, let us theoretically acquire the BIC position using the multipolar expansion technique [8, 9, 10, 4]. If we again assume a square array composed of identical and isotropic particles (Fig. S1), its response to an incident field can be described by substituting (1)-(3) from the Main article into (S3), and, thus, a linear system of equations is formed. Solving the eigenvalue problem leads to the modes of the array, including, in this case, the trapped ones that do not couple with radiation channels, i.e., the BICs. Therefore, if we invert the square matrix of the system to the left side and set the excitation to zero, or $\mathbf{q}^{\{\mathrm{e,m}\}} = \mathbf{0}$, the resulting homogenous system will have a non-trivial solution if the determinant of the matrix is zero. In particular, after some algebra, the BIC condition is reduced to,

$$\left| \bar{\bar{I}} - \bar{\bar{T}}_0 \,\bar{\bar{C}}_s \right| = 0. \tag{S12}$$

The equation above is general in nature and can be used for any type of lattice or particle in a homogenous medium [4]. Due to its complexity, (S12) can only be solved numerically in its general form, i.e. for higher order multipoles or more diverse lattices. Nevertheless, for specific reduced cases, versatile analytic solutions can be found, as demonstrated in [4] for the case of the coupled electric dipole - magnetic quadrupole on a square lattice and for a TM-polarized plan wave at normal incidence.

The matrix $\bar{\bar{C}}_s$ is the *lattice coupling matrix* expressed in spherical coordinates, which is a function of the unit cell dimension, the frequency, and the wavevector, and can be calculated via rapidly converging summations using Ewald's method [3, 11, 12].

In this work, we consider a square lattice decorated by isotropic and lossless particle whose response is expanded only up to dipolar order, or $j = 1$. Then, because the elements of the lattice interaction matrix, $\bar{\bar{C}}_s$, can be pre-calculated for a specific incident wavevector, $\mathbf{k}^{\mathrm{inc}}$, and a normalized lattice dimension, $d/\lambda$, eventually, (S12) can be solved with the Mie angles of (S7) for $j = 1$ as the unknowns. Specifically, for a dipole approximation and an oblique incidence on the lattice, $\bar{\bar{C}}_s$ is simplified to,

$$\bar{\bar{C}}_s = \begin{bmatrix} C^{\mathrm{ee}}_{-1-1} & 0 & C^{\mathrm{ee}}_{-11} & 0 & C^{\mathrm{em}}_{-10} & 0 \\ 0 & C^{\mathrm{ee}}_{00} & 0 & C^{\mathrm{em}}_{0-1} & 0 & C^{\mathrm{em}}_{01} \\ C^{\mathrm{ee}}_{1-1} & 0 & C^{\mathrm{ee}}_{11} & 0 & C^{\mathrm{em}}_{10} & 0 \\ 0 & C^{\mathrm{me}}_{-10} & 0 & C^{\mathrm{mm}}_{-1-1} & 0 & C^{\mathrm{mm}}_{-11} \\ C^{\mathrm{me}}_{0-1} & 0 & C^{\mathrm{me}}_{01} & 0 & C^{\mathrm{mm}}_{00} & 0 \\ 0 & C^{\mathrm{me}}_{10} & 0 & C^{\mathrm{mm}}_{1-1} & 0 & C^{\mathrm{mm}}_{11} \end{bmatrix} = \begin{bmatrix} C_1 & 0 & C_3 & 0 & C_5 & 0 \\ 0 & C_2 & 0 & C_5 & 0 & C_5 \\ C_3 & 0 & C_1 & 0 & C_5 & 0 \\ 0 & C_5 & 0 & C_1 & 0 & C_3 \\ C_5 & 0 & C_5 & 0 & C_2 & 0 \\ 0 & C_5 & 0 & C_3 & 0 & C_1 \end{bmatrix}. \tag{S13}$$

Let us now assume an oblique TE (or s-polarized) incident wave. For this specific incidence, a dipole approximation and an isotropic particle only the $m_x$, $p_y$, and $m_z$ dipoles are excited, or, if the spherical coordinates are used, the T matrix turns to $\bar{\bar{T}}_0 = \mathrm{diag}(-a_1, 0, -a_1, -b_1, -b_1, -b1)$. Therefore, (S12) turns to

$$\begin{aligned} &[(C_1 - C_3)a_1 + 1] \cdot \left[(C_1^2 - C_3^2)b_1^2 + 2C_1 b_1 + 1\right] \cdot \\ &\qquad \left[(C_1 C_2 + C_2 C_3 - 2C_5^2)a_1 b_1 + (C_1 + C_3)a_1 + C_2 b_1 + 1\right] = 0. \end{aligned} \tag{S14}$$

The first and second terms are associated with collective lattice resonances, which refer to the collective response of the 2D identical particle array [13, 14, 4, 15]. These resonances exhibit no BIC behavior as they radiate in the environment, and their study is out of the scope of this work. The third term describes the coupling of the $p_y$ and $m_z$ dipoles and, since the accidental BICs rely on the interference between in-plane and out-of-plane multipoles to suppress the far-field radiation, it will provide the condition necessary for the appearance of the BIC mode (see Fig. 2(a)). The same procedure can be repeated for the TM (or p-polarized) incidence, but this time only the $p_x$, $m_y$ and $p_z$ dipoles are excited. Thus, one must insert $\bar{\bar{T}}_0 = \mathrm{diag}(-a_1, -a_1, -a_1, -b_1, 0, -b1)$ in (S12).

Following the procedure above, the relation between the dipolar magnetic and electric Mie coefficients is derived for TE or TM incident wave, respectively, from (S12) as,

$$b_1 = -\frac{1 + a_1 \left(C_1 + C_3\right)}{C_2 + a_1 \left(C_1 C_2 + C_2 C_3 - 2\, C_5^2\right)}, \quad \text{(TE / s-polarized incidence)} \tag{S15a}$$

$$a_1 = -\frac{1 + b_1 \left(C_1 + C_3\right)}{C_2 + b_1 \left(C_1 C_2 + C_2 C_3 - 2\, C_5^2\right)} \quad \text{(TM / p-polarized incidence).} \tag{S15b}$$

where $C_1$, $C_2$, $C_3$, and $C_5$, are the dipole-dipole interaction coefficients or the elements of the $\bar{\bar{C}}_s$ for $j = 1$ for a square lattice [3, 4], as demonstrated in (S13). Note that the equations for TE and TM equations are similar with only the coefficients $a_1$ and $b_1$ swapped, as anticipated due to symmetry. One should notice from (S15) that the electric-magnetic lattice coupling coefficient is crucial for the existence of a $(a_1, b_1)$ solution, indicating the importance of multipolar, electromagnetic coupling for realizing a BIC. Hence, for the specific aforementioned cases, (S15) gives the exact position of the BIC for given TE and TM incidences, respectively, and they will greatly aid the design and post-processing analysis, herein. In this context, it will become evident why the Mie angle representation of the Mie coefficients is convenient for the identification of the BIC points. For example, for the s-polarized incidence case, by substituting (S7) into (S15a), and due to the boundaries of $\theta_{\mathrm{E1}}$ and $\theta_{\mathrm{M1}}$ between $-\pi/2$ and $\pi/2$, the exact solution can be easily obtained via a non-linear equation solver for a given wavelength and lattice dimension. Alternatively, one can sweep $\theta_{\mathrm{E1}}$ in (S15a) between between $-\pi/2$ and $\pi/2$ and keep only the solutions that satisfy the condition $|b_1| = 1$ for lossless particles[5], as performed in [4].

# 6 Computation of the lattice coupling matrix $\bar{\bar{C}}_s$

In this section, we briefly provide the formulas to calculate the elements of a lattice coupling matrix for a 2D multipole array. This discussion is entirely based on our previous work [3, 4, 11]. The lattice coupling matrix $\bar{\bar{C}}_s$ depends on the dimensions of the 2D array and the relative phase between lattice sites expressed by the components of wave vector $\mathbf{k}_{||}$ of the incident plane wave that are tangential with respect to the lattice. The vectors $\mathbf{R} = n_1\mathbf{u}_1 + n_2\mathbf{u}_2$, $n_1, n_2 \in \mathbb{Z}$ cover all lattice sites with the vectors $\mathbf{u}_1$ and $\mathbf{u}_2$ describing one unit cell of the lattice. We focus now at the particle at the origin. The response of all other sites can be obtained by including the correct phase. The matrix $\bar{\bar{C}}_s$ is calculated after summating the translation coefficients from all other positions on the 2D array to $\mathbf{R} = 0$. The radiated fields from a

point $\mathbf{R}$ can be reexpanded at the origin using the translation properties of the VSHs [16]

$$\mathbf{M}^{(3)}_{jm}(k\mathbf{r}-k\mathbf{R}) = \sum_{\iota=1}^{\infty}\sum_{\mu=-\iota}^{\iota} A_{\iota\mu jm}(-k\mathbf{R})\mathbf{M}^{(1)}_{\iota\mu}(k\mathbf{r}) + B_{\iota\mu jm}(-k\mathbf{R})\mathbf{N}^{(1)}_{\iota\mu}(k\mathbf{r}), \tag{S16}$$

$$\mathbf{N}^{(3)}_{jm}(k\mathbf{r}-k\mathbf{R}) = \sum_{\iota=1}^{\infty}\sum_{\mu=-\iota}^{\iota} B_{\iota\mu jm}(-k\mathbf{R})\mathbf{M}^{(1)}_{\iota\mu}(k\mathbf{r}) + A_{\iota\mu jm}(-k\mathbf{R})\mathbf{N}^{(1)}_{\iota\mu}(k\mathbf{r}), \tag{S17}$$

expressed by the translation coefficients

$$A_{\iota\mu jm}(kr,\theta,\phi) = \frac{\gamma_{jm}}{\gamma_{\iota\mu}}(-1)^m \frac{2\iota+1}{\iota(\iota+1)}\mathrm{i}^{\iota-j}\sqrt{\pi\frac{(j+m)!(\iota-\mu)!}{(j-m)!(\iota+\mu)!}}\sum_p \mathrm{i}^p\sqrt{2p+1}h^{(1)}_p(kr)Y_{p,m-\mu}(\theta,\phi)\times \begin{pmatrix} j & \iota & p \\ m & -\mu & -m+\mu \end{pmatrix}\begin{pmatrix} j & \iota & p \\ 0 & 0 & 0 \end{pmatrix}\left[j(j+1)+\iota(\iota+1)-p(p+1)\right], \tag{S18a}$$

$$B_{\iota\mu jm}(kr,\theta,\phi) = \frac{\gamma_{jm}}{\gamma_{\iota\mu}}(-1)^m \frac{2\iota+1}{\iota(\iota+1)}\mathrm{i}^{\iota-j}\sqrt{\pi\frac{(j+m)!(\iota-\mu)!}{(j-m)!(\iota+\mu)!}}\sum_p \mathrm{i}^p\sqrt{2p+1}h^{(1)}_p(kr)Y_{p,m-\mu}(\theta,\phi)\times \begin{pmatrix} j & \iota & p \\ m & -\mu & -m+\mu \end{pmatrix}\begin{pmatrix} j & \iota & p-1 \\ 0 & 0 & 0 \end{pmatrix}\sqrt{\left[(j+\iota+1)^2-p^2\right]\left[p^2-(j-\iota)^2\right]}. \tag{S18b}$$

with the Wigner 3j-symbols $\begin{pmatrix} j_1 & j_2 & j_3 \\ m_1 & m_2 & m_3 \end{pmatrix}$ [17, 18]. The sum index $p$ takes all integer values for which the Wigner 3j-symbols are non-zero. The normalization factor $\gamma$ is given in (S2c), $h^{(1)}_p(x)$ are the spherical Hankel function of the first kind and $Y_{p,m-\mu}(\theta,\rho)$ are the spherical harmonics. Expressed in matrix form and summing up the response of all other lattice sites to $\mathbf{R}=0$, $\bar{\bar{C}}_s$ is defined as

$$\bar{\bar{C}}_s = \sum_{\mathbf{R}\neq 0}\begin{pmatrix} \bar{\bar{A}}(-k\mathbf{R}) & \bar{\bar{B}}(-k\mathbf{R}) \\ \bar{\bar{B}}(-k\mathbf{R}) & \bar{\bar{A}}(-k\mathbf{R}) \end{pmatrix} e^{\mathrm{i}\mathbf{k}_\parallel\cdot\mathbf{R}}, \tag{S19}$$

with the rows and columns of $\bar{\bar{A}}$ and $\bar{\bar{B}}$ given by the translation coefficients with $j,\iota\in\mathbb{N}$, $m\in\{-j,-j+1,\dots,j\}$, and $\mu\in\{-\iota,-\iota+1,\dots,\iota\}$. Now, the crucial step for the numerical evaluation of the matrix coeffiecients is the lattice sum over $\mathbf{R}$. The part of the $C_s$ elements that depends on $R$ is given by

$$D_{jm} = \sum_{\mathbf{R}\neq 0} h^{(1)}_j(kR)Y_{jm}\left(\theta_{-\mathbf{R}},\phi_{-\mathbf{R}}\right)e^{\mathrm{i}\mathbf{k}_\parallel\mathbf{R}}. \tag{S20}$$

The direct evaluation of (S20) is generally converging extremely slowely. Therefore, we make use of the *Ewald summation method* [19]. By separating short- and long-range contributions, we can sum them independently in real and Fourier space. This separation leads to two separate quickly converging series. Conventionally writing these parts as $D_{jm} = D^{(1)}_{jm} + D^{(2)}_{jm} + D^{(3)}_{jm}$, where the third summand includes a correction term for the missing origin contribution when converting the long-range part to Fourier space,

we get by following [20] the expressions

$$D_{jm}^{(1)} = \frac{\sqrt{(2j+1)(j-m)!(j+m)!}}{Ak\,\mathrm{i}^{-m}} \sum_{\mathbf{G}} \left(\frac{|\mathbf{k}_\parallel + \mathbf{G}|}{2k}\right)^j \frac{e^{\mathrm{i}m\phi_{\mathbf{k}_\parallel+\mathbf{G}}}}{k_{\mathbf{G},z}^{+}} \sum_{\lambda=0}^{\frac{j-|m|}{2}} \frac{\left(\frac{k_{\mathbf{G},z}^{+}}{|\mathbf{k}_\parallel+\mathbf{G}|}\right)^{2\lambda} \Gamma\left(\frac{1}{2}-\lambda, -\frac{(k_{\mathbf{G},z}^{+})^2}{4T^2}\right)}{\lambda!\left(\frac{j+m}{2}-\lambda\right)!\left(\frac{j-m}{2}-\lambda\right)!}, \tag{S21a}$$

$$D_{jm}^{(2)} = \frac{-\mathrm{i}(-1)^{\frac{j+m}{2}}\sqrt{(2j+1)(j-m)!(j+m)!}}{2^{j+1}\pi\frac{j-m}{2}!\frac{j+m}{2}!} \sum_{\mathbf{R}\neq 0} e^{\mathrm{i}\mathbf{k}_\parallel\cdot\mathbf{R}+\mathrm{i}m\phi_{-\mathbf{R}}} \frac{1}{k}\left(\frac{2R}{k}\right)^j \int_{T^2}^{\infty} u^{j-\frac{1}{2}} e^{-R^2u+\frac{k^2}{4u}}\mathrm{d}u, \tag{S21b}$$

$$D_{jm}^{(3)} = \frac{\delta_{j0}}{4\pi}\Gamma\left(-\frac{1}{2}, -\frac{k^2}{4T^2}\right). \tag{S21c}$$

The $D_{jm}^{(1)}$ term involves the reciprocal lattice $\mathbf{G}$ and the wavenumber $k_{\mathbf{G},z}^{+}$ explained in (S4), while $\Gamma(\frac{1}{2}-\lambda, z)$ is the the upper incomplete Gamma function. The parameter $T$ is giving the separation between the real and the Fourier space summation [3] and $A$ is the area of the unit cell defined by $\mathbf{u}_1$ and $\mathbf{u}_2$. Finally, the integral in $D_{jm}^{(2)}$ is calculated using a recursion relation, while the $\delta_{ij}$ in $D_{jm}^{(3)}$ is the Kronecker delta. After calculating $D_{jm}$ of (S21) with these expressions, one can insert the retrieved value in (S19) and, thus, obtaining the desired lattice coupling matrix $\bar{\bar{C}}_s$.

# 7 Two BICs in one band

During the analysis of BIC robustness in k-space, we found that there is a combination of Mie angles at which two BICs on the same mode line can exist. Figure. S3(a) shows the BIC positions for different angles $\theta$ and frequencies $\tilde{\omega} = \omega d/2\pi c$. It turns out that there are BICs in different points of k-space that are characterized by the same optical response of a single scatterer, which suggests that these two BICs exist on the same modal line. The first BIC is for $\theta_1 = 43°$ and $\tilde{\omega} = 0.501$, the second BIC is for $\theta_1 = 52.5°$ and $\tilde{\omega} = 0.466$. There are two points on the blue and orange line for which the combination of Mie angles is the same (see Fig. S3(b)). The Mie angles for this example are $\theta_{E1} = -0.915171, \theta_{M1} = 0.066822$. Fig. S3(c) shows the reflection $R$ as a function of the normalized frequency $\omega d/2\pi c$ and the incident angle $\theta = 42° - 54°$ for the square lattice with period $d = 450\,\mathrm{nm}$ and demonstrates that two BICs can exist on one mode line. The green and purple circles mark the areas where the BICs appear. Fig. S3(b) shows the dependence of the Q-factor on the angle of incidence $\theta$ and how it changes dramatically for angles $\theta = 43°$ and $\theta = 52.5°$.

# References and Notes


[1] M. I. Mishchenko, L. D. Travis, and A. A. Lacis, *Scattering, absorption, and emission of light by small particles*, Cambridge university press (2002).

[2] T. Antonakakis, F. I. Baida, A. Belkhir, *et al.*, "Gratings: Theory and numeric applications," (2014).

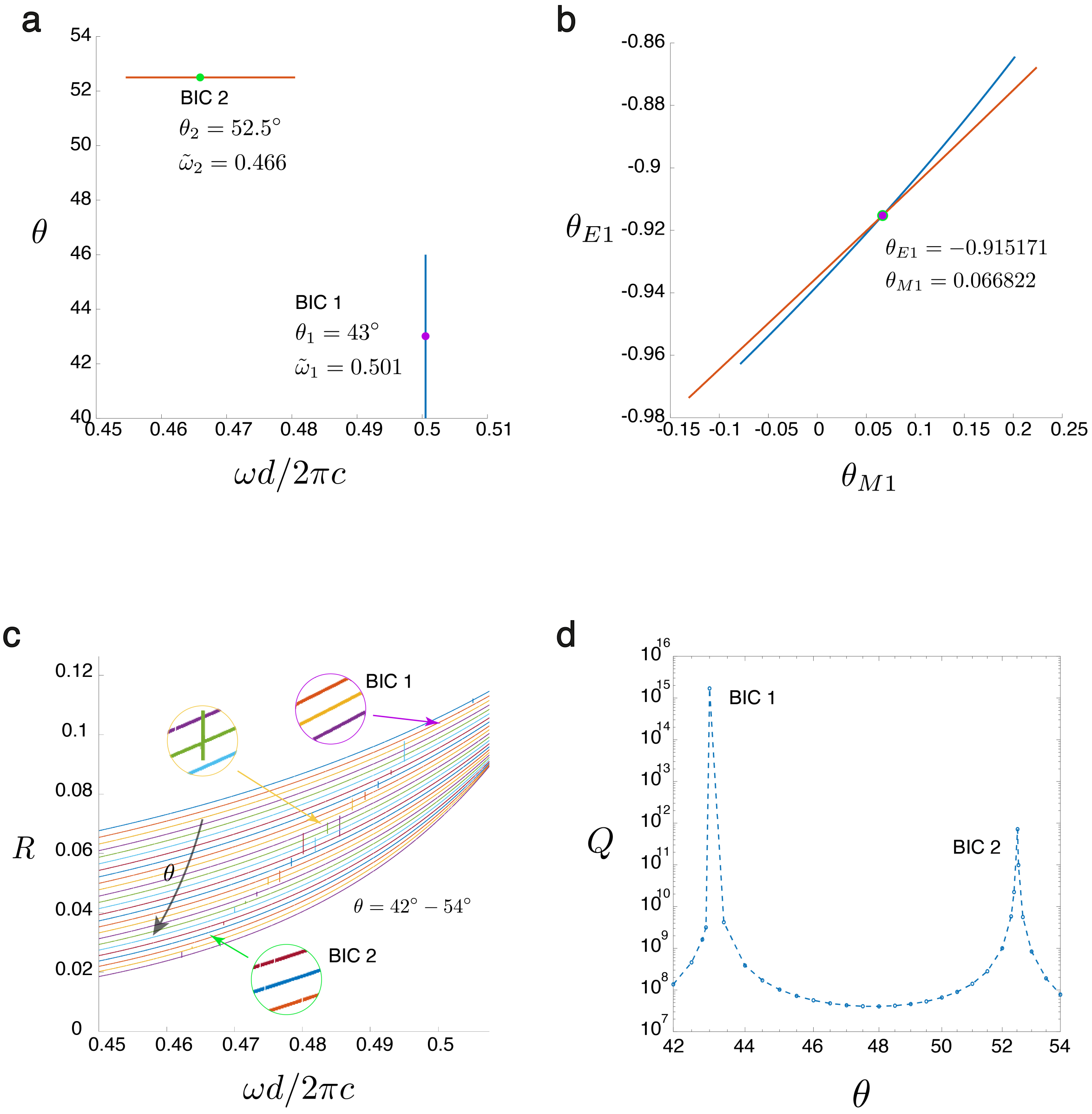


Figure S3: Two BICs on one mode line. (a) the positions of the BICs for different angles $\theta$(deg) and frequency $\tilde{\omega} = \omega d/2\pi c$ values: first BIC is for $\theta_1 = 43\circ$ and $\tilde{\omega} = 0.501$, second BIC is for $\theta_1 = 52.5°$ and $\tilde{\omega} = 0.466$. (b) the values of the Mie angles for these BICs from panel a. (c) The reflection R as a function of dimensionless frequency $\omega d/2\pi c$ and angle of incidence $\theta = 42° - 54°$ for a square lattice with period d = 450 nm. (d) The Q-factor as a function of the angle of incidence $\theta$.

[3] D. Beutel, A. Groner, C. Rockstuhl, *et al.*, "Efficient simulation of biperiodic, layered structures based on the t-matrix method," *Journal of the Optical Society of America B* **38**(6), 1782–1791

(2021).

[4] A. Rahimzadegan, T. D. Karamanos, R. Alaee, *et al.*, "A comprehensive multipolar theory for periodic metasurfaces," *Advanced Optical Materials* **10**(10), 2102059 (2022).

[5] A. Rahimzadegan, R. Alaee, C. Rockstuhl, *et al.*, "Minimalist mie coefficient model," *Optics Express* **28**(11), 16511–16525 (2020).

[6] A. Rahimzadegan, R. Alaee, T. Karamanos, *et al.*, "Colossal enhancement of the magnetic dipole moment by exploiting lattice coupling in metasurfaces," *JOSA B* **38**(9), C217–C224 (2021).

[7] J. Mun, S. So, J. Jang, *et al.*, "Describing meta-atoms using the exact higher-order polarizability tensors," *ACS Photonics* **7**(5), 1153–1162 (2020).

[8] K. Koshelev and Y. Kivshar, "Dielectric resonant metaphotonics," *ACS Photonics* **8**(1), 102–112 (2020).

[9] D. R. Abujetas, J. Olmos-Trigo, J. J. Sáenz, *et al.*, "Coupled electric and magnetic dipole formulation for planar arrays of particles: Resonances and bound states in the continuum for all-dielectric metasurfaces," *Physical Review B* **102**(12), 125411 (2020).

[10] A. B. Evlyukhin, V. R. Tuz, V. S. Volkov, *et al.*, "Bianisotropy for light trapping in all-dielectric metasurfaces," *Physical Review B* **101**(20), 205415 (2020).

[11] D. Beutel, I. Fernandez-Corbaton, and C. Rockstuhl, "Unified lattice sums accommodating multiple sublattices for solutions of the helmholtz equation in two and three dimensions," *Physical Review A* **107**(1), 013508 (2023).

[12] I. M. Fradkin, S. A. Dyakov, and N. A. Gippius, "Fourier modal method for the description of nanoparticle lattices in the dipole approximation," *Physical Review B* **99**(7), 075310 (2019).

[13] F. G. De Abajo, R. Gómez-Medina, and J. Sáenz, "Full transmission through perfect-conductor subwavelength hole arrays," *Physical review E* **72**(1), 016608 (2005).

[14] A. D. Utyushev, V. I. Zakomirnyi, and I. L. Rasskazov, "Collective lattice resonances: Plasmonics and beyond," *Reviews in Physics* **6**, 100051 (2021).

[15] D. R. Abujetas, J. Olmos-Trigo, and J. A. Sánchez-Gil, "Tailoring accidental double bound states in the continuum in all-dielectric metasurfaces," *Advanced Optical Materials* , 2200301 (2022).

[16] L. Tsang, J. A. Kong, and R. T. Shin, *Theory of Microwave Remote Sensing (Wiley Series in Remote Sensing and Image Processing)*, Wiley-Interscience (1985).

[17] E. P. Wigner, "On the matrices which reduce the kronecker products of representations of sr groups," in *The Collected Works of Eugene Paul Wigner*, 608–654, Springer (1993).

[18] A. Messiah, "Clebsch-gordan (c.-g.) coefficients and'3j'symbols," *Appendix CI in Quantum Mechanics* **2**, 1054–1060 (1962).

[19] P. P. Ewald, “Die berechnung optischer und elektrostatischer gitterpotentiale,” *Annalen der Physik* **369**(3), 253–287 (1921).

[20] K. Kambe, “Theory of Low-Energy Electron Diffraction (I. Application of the Cellular Method to Monatomic Layers),” *Zeitschrift für Naturforschung A* **22** (1967).
